# Glucose deprivation and identification of TXNIP as an immunometabolic modulator of T cell activation in cancer

**DOI:** 10.3389/fimmu.2025.1548509

**Published:** 2025-04-07

**Authors:** Agathe Dubuisson, Adèle Mangelinck, Samantha Knockaert, Adrien Zichi, Etienne Becht, Wendy Philippon, Sandra Dromaint-Catesson, Manon Fasquel, Fabien Melchiore, Nicolas Provost, Dawid Walas, Hélène Darville, Jean-Pierre Galizzi, Céline Lefebvre, Véronique Blanc, Vincent Lombardi

**Affiliations:** ^1^ Servier, Research and Development, Gif-sur-Yvette, France; ^2^ Faculty of Medicine, University of Opole, Opole, Poland

**Keywords:** tumor microenvironment, cancer immunotherapy, glucose deprivation, T cells activation, TXNIP, mixed lymphocyte reaction, single-cell RNA-sequencing

## Abstract

**Background:**

The ability of immune cells to rapidly respond to pathogens or malignant cells is tightly linked to metabolic pathways. In cancer, the tumor microenvironment (TME) represents a complex system with a strong metabolism stress, in part due to glucose shortage, which limits proper T cell activation, differentiation and functions preventing anti-tumor immunity.

**Methods:**

In this study, we evaluated T cell immune reactivity in glucose-restricted mixed lymphocyte reaction (MLR), using a comprehensive profiling of soluble factors, multiparametric flow cytometry and single cell RNA sequencing (scRNA-seq).

**Results:**

We determined that glucose restriction potentiates anti-PD-1 immune responses and identified thioredoxin-interacting protein (TXNIP), a negative regulator of glucose uptake, as a potential immunometabolic modulator of T cell activation. We confirmed TXNIP downregulation in tumor infiltrating T cells in cancer patients. We next investigated the implication of TXNIP in modulating immune effector functions in primary human T cells and showed that TXNIP depletion increased IFN-γ secretion and tumor cell killing.

**Conclusions:**

TXNIP is at the interface between immunometabolism and T cell activation and could represent a potential target for immuno-oncology treatments.

## 1 Introduction

Cancer cells undergo metabolic reprogramming characterized by high glucose uptake from the extracellular environment and aerobic glycolysis. This altered metabolism, known as the Warburg effect, allows cancer cells to meet the energy requirements for their abnormal proliferation (1, 2). Consequently, solid tumor microenvironment (TME) displays specific and drastic conditions, including nutrients deprivation like glucose starvation. Importantly, glucose is not only essential for cancer cell metabolism, but also for immune cells in the TME. Glucose competition between cancer and immune cells can profoundly impact the latter’s ability to survive, proliferate and generate an efficient immune response against the tumor (3). Thus, understanding the complex interplay between glucose metabolism and the immune response in the TME is crucial for the development of effective cancer immunotherapy.

The mixed lymphocyte reaction (MLR) is an *in vitro* cellular assay modeling T cell activation during an allogeneic immune reaction (4). It is a powerful tool that engages the whole immunological synapse and mimics complex physiological T cell responses. This assay is widely used in drug discovery to evaluate the immunomodulatory effects of new compounds and has been used for the *in vitro* evaluation and validation of nivolumab activity, an anti-PD-1 monoclonal antibody (mAb) indicated for the treatment of many cancer types (5, 6).

Thioredoxin-interacting protein (TXNIP), also known as Thioredoxin-binding protein-2 or Vitamin D3 up-regulated protein-1, is commonly considered an endogenous antagonist of thioredoxin (TRX), a key regulator in cellular redox balance (7, 8). It is an α-arrestin protein acting as an oxidative stress mediator by inhibiting TRX activity. TXNIP has also been implicated in multiple other biological processes including the negative regulation of glucose uptake and metabolism notably through facilitating glucose transporter 1 (GLUT1) and glucose transporter 4 (GLUT4) endocytosis (9–13) as well as cell proliferation and cell division by repressing cell cycle regulatory proteins such as p27^kip1^, JAB1, Cdk2 and cyclin E (14).

TXNIP has been extensively studied in metabolic disorders such as diabetes and obesity, where it plays a role in the apoptosis of pancreatic β cells (15). In cancer, TXNIP was initially considered a tumor suppressor, this being notably supported by the observation that TXNIP-deficient mice exhibit a 40% higher incidence of spontaneously developing hepatocellular carcinoma (9, 15). However, different studies have mitigated this initial assumption, suggesting that the role of TXNIP is complex in cancer (reviewed in (16)). Interestingly, in human, the absence of TXNIP in a family led to lactic acidosis and diabetes but no cancer was reported (17). TXNIP was studied in various immunological contexts, including chronic inflammation, viral infection, and immunization using mouse models. It plays a role in natural killer (NK) cell development and functions, without affecting the development or homeostasis of T and B cells (18–20). In T cells more specifically, TXNIP is proposed to be implicated in T cell proliferation, glucose uptake and IFNG regulation (19, 21–24) but no other studies have explored in more details the role of TXNIP in T cells. To our knowledge, the impact of TXNIP in human T cell effector functions in the context of immuno-oncology has never been reported.

In this study, to assess the effect of glucose restriction on T cell responses, we developed an MLR model mimicking the *in situ* glucose concentration found in most TME (*i.e.* 1 mM or low glucose, LG condition), compared to conventional *in vitro* glucose level used in culture condition (*i.e.* 11 mM or high glucose, HG condition). This glucose-deprived MLR assay allowed us to investigate the effect of low glucose on immune effector functions in primary human T cells. We identified TXNIP to be down-regulated both upon CD4^+^ T cell stimulation and glucose deprivation. In parallel, patients’ data were analyzed and showed that TXNIP is highly expressed in T lymphocytes and that its expression is reduced in tumor-infiltrating CD4^+^ T cells compared to those in paired normal adjacent tissue and blood. Next, we proposed that TXNIP acts as an inhibitor of IFN-γ production and demonstrated by CRISPR Cas-9 genome editing that TXNIP depletion can enhance T cell effector functions.

## 2 Materials and methods

### 2.1 Statistical analysis

Statistical analyses were conducted in R (v4.3.1).

For flow cytometry and cytokine secretion data, Shapiro test was performed to assess the distribution of data, and Student’s t-test or Wilcoxon test were used to calculate the significance between two means.

For glycolysis score analysis on single cell data, one-way analysis of variance (ANOVA) with Tukey’s multiple comparisons tests were performed using the multcomp package (v1.4-25) for multiple group comparisons.

For all tests, an adjusted p-value inferior to 0.05 was considered statistically significant.

### 2.2 Study approval

The studies involving humans were approved by Ethics committee of Etablissement Français du Sang (EFS), Ile-de-France (agreement n°21/EFS/035). The studies were conducted in accordance with the local legislation and institutional requirements. The participants provided their written informed consent to participate to this study.

### 2.3 Isolation of primary cells (PBMC, CD14^+^ monocytes, CD4^+^ T cells and PBT)

Blood samples from healthy donors were purchased from Etablissement Français du Sang (EFS, Pontoise, France). Sex was not considered as a biological variable. Peripheral blood mononuclear cells (PBMCs) were isolated from buffy coat by density gradient centrifugation using Ficoll: Lymphoprep (cat no. 07801, StemCell). CD14^+^ and CD4^+^ populations as well as peripheral blood T cells (PBT) were purified from healthy donors’ PBMCs using magnetic CD14 isolation beads by positive selection (CD14 Microbeads human, cat no. 130-050-201, Miltenyi), CD4 (CD4 T Cell Isolation kit, cat no. 130-096-533, Miltenyi) isolation beads by negative selection, or collecting the negative fraction of magnetic CD14 isolation beads, respectively, following the manufacturer’s instructions.

### 2.4 MoDC generation from CD14^+^ cells

Monocyte-derived Dendritic cells (MoDC) were generated by culturing CD14^+^ monocytes (10^6^/mL) isolated from PBMCs of healthy donors for 7 days with MoDC media: RPMI 1640 GlutaMAX (cat no. 61870, Gibco) supplemented with 10% heat inactivated serum (cat no. CVSVF00-01, Eurobio), 1% P/S (cat no. 15140, Gibco), HEPES 10 mM (cat no. 15630, Gibco), 50 ng/mL interleukin-4 (IL-4, cat no. 130-093-922, Miltenyi) and 100 ng/mL GM-CSF (cat no. 130-093-866, Miltenyi) (25). On day 4, 20% of MoDC media was added to the flasks.

After 7 days, and prior to co-culture, MoDC were tested for maturation status by looking at CD1a, CD83, CD86, Tim-3, PD-L1 and HLA-DR expression by flow cytometry.

### 2.5 One-way mixed lymphocyte reaction

**Figure 1:**
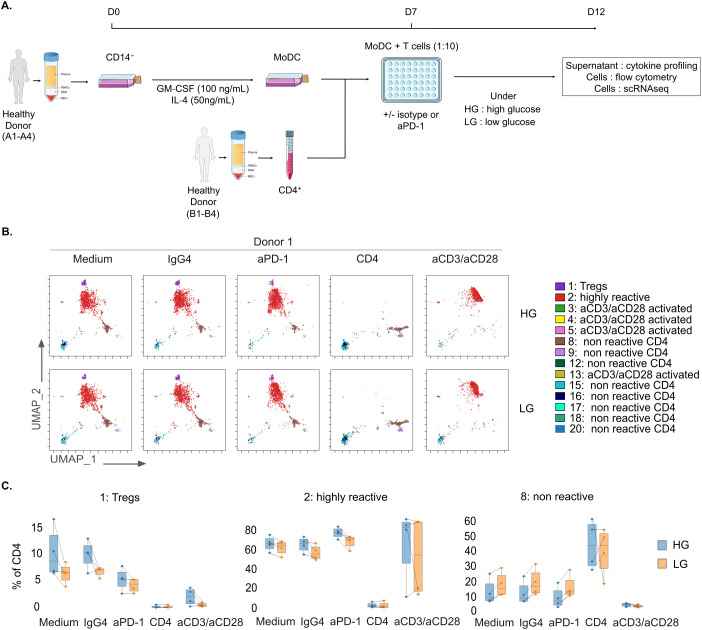
Impact of glucose deprivation on CD4^+^ T cell immunoreactivity. **(A)** Schematic representation of the MLR protocol for glucose deprivation study. **(B)** Representative flow cytometry UMAP dimensionality reduction representation of CD4^+^ T cells upon MLR stimulation colored by non-supervised clustering (Donor 1, n=1). **(C)** Box plots of CD4^+^ T cells proportions in Tregs (left panel), highly reactive (middle panel), or non-reactive (right panel) clusters, displaying group of numerical data through their 3^rd^ and 1^st^ quantiles (box), median (central band), minimum and maximum (whiskers) (n=4). HG, High Glucose (11 mM); LG, Low Glucose (1mM).

For glucose deprivation study in MLR, freshly isolated CD4^+^ T cells (10^5^ cells) and allogeneic MoDCs (10^4^ cells) were co-cultured in triplicate in ultra-low attachment 96-well microplates (Corning, cat no. 7007) in culture media consisting of RPMI 1640 no Glucose (cat no. 11879, Gibco) supplemented with 10% heat inactivated serum (cat no. CVSVF00-01, Eurobio), 1% Penicillin/Streptomycin (P/S, cat no. 15140, Gibco) as well as either 11mM D-Glucose or 1mM D-Glucose (cat no. G8769, Sigma), resulting in high glucose (HG) or low glucose (LG) media, respectively. Four distinct MLRs were performed (see **Figure 1A**). For each MLR, cells were either left untreated (medium), treated with isotype control (IgG4) or anti-PD-1 (nivolumab). CD4^+^ T cells alone or stimulated with anti-CD3/anti-CD28 antibodies (T cell activator, cat no. 10971, StemCell) were used as negative and positive controls, respectively. On day 5, culture supernatants and cells were collected. Culture supernatants were immediately frozen at -80°C for at least 24h, until further analyses. Cells were collected and resuspended for either flow cytometry or scRNA-seq analyses. Cytokine release and flow cytometry were performed on all samples whereas scRNA-seq was executed on one set of MLR (using Donor 1: A1+B1). Five other sets of MLRs under HG or LG conditions were used for assessing TXNIP expression at the protein level. To this end, cells were collected on day 5 and lysed for western blot analyses.

For the functional evaluation of TXNIP in MLR, CD4^+^ T cells (10^5^ cells) following TXNIP CRISPR-Cas9 genome-editing knockout (TXNIP KO) and allogeneic MoDCs (10^4^ cells) were co-cultured in triplicate in ultra-low attachment 96-well microplates (Corning, cat no. 7007) in complete RMPI Glutamax culture media consisting of RPMI 1640 GlutaMAX (cat no. 61870, Gibco) supplemented with 10% heat inactivated serum (cat no. CVSVF00-01, Eurobio) and 1% P/S (cat no. 15140, Gibco). Six different MLRs were performed. For each MLR, cells were either left untreated (medium), treated with isotype control (IgG4) or anti-PD-1 (nivolumab). TXNIP KO CD4^+^ T cells alone or stimulated with anti-CD3/anti-CD28 antibodies (T cell activator, cat no. 10971, StemCell) were used as negative and positive control, respectively. On day 6, culture supernatants and cells were collected. Culture supernatants were immediately frozen at -80°C for at least 24h, until further analyses. Cells were collected and resuspended for flow cytometry analyses.

### 2.6 Multiplex cytokine and chemokine assays

For glucose deprivation study in MLR (n=4), samples were thawed and monitored in triplicates for cytokine and chemokine content using Milliplex MAP Human TH17 kit (Merck, cat no. HCYTOMAG-60K). Acquisition and analyses were performed on a Bio-Plex 200 system (Bio-Rad) and the Bio-Plex Manager 6.1 Software (Bio-Rad).

For the functional evaluation of TXNIP in MLR (n=6), samples were thawed and monitored in triplicate for cytokine and chemokine content using U-plex set (cat no. K15067M-2, Meso Scale Discovery) following manufacturer’s instructions. Acquisition and analyses were performed on a MESO™ QuickPlex SQ120 reader and the MSD’s Discovery Workbench 4.0 software.

Data visualization and calculations were performed with R (v4.3.1) using dplyr (v1.1.4), tidyr (v1.3.1), ggpubr (v0.6.0), and Hmisc (v5.1.1) packages. All calculations were performed in R v4.3.1.

### 2.7 Flow cytometry

MoDCs were tested for maturation status using anti-CD14-AF488 (clone: M5E2, Biolegend), anti-CD3-PercP (clone: UCHT1, Biolegend), anti-CD1a-APC (clone: HI149, Miltenyi), anti-CD83-BV605 (clone: HB15, Biolegend), anti-CD86-PE/Vio770 (clone: FM95, Miltenyi), anti-HLA-DR-BV610 (clone: L243, Biolegend), anti-Tim-3-BV711 (clone: F38-E2E, Biolegend), anti-OX40-L-PE (clone: ANC10G1, Ancell), anti-PD-1-BV421 (clone: EH12.2H7, Biolegend), and anti-PD-L1-PECF594 (clone: 2A3, Biolegend) antibodies. Viability was assessed using Zombie NIR (cat no. 423106, Biolegend). Acquisition and analyses were performed on CytoFLEX S (Beckman Coulter).

For glucose deprivation study in MLR (n=4), cells were labeled to measure T cell proliferation and activation status after 5 days of co-culture. Fc receptor blocking was performed on the total cell suspension using human FcR Blocking reagent (cat no. 130-059-901, Miltenyi). Cell viability was performed using Maleimide (cat no. 1408, AAT Bioquest). For cell surface phenotyping, antibodies specific for CD3-APC-Cy7, CD4-BUV496, CD8-PerCP, CCR7-BV510, CD45RA-BV805, CD25-FITC, CTLA-4-PECF594, PD-1-BUV737, Tim-3-BV785, LAG-3-BV421, ICOS-AF700, TIGIT-BUV595, and NKG2A-PC7 were used. Cell suspensions were subsequently stained for intracellular phenotyping: cells were fixed and permeabilized using human FoxP3/Transcription Factor Staining Buffer Set (cat no. 00-5523-00, Thermo Fisher Scientific) according to the manufacturer’s directions and stained with FoxP3-PE (clone: PCH101, eBioscience), TOX-APC (clone: TXRX10, eBioscience) and Ki67-BV711 (cat no. 350516, BioLegend). Cells were acquired on CytoFLEX LX (Beckman Coulter). Data visualization and analysis were performed using Kaluza Analysis software v2.1 (Beckman Coulter), Cytobank software (Beckman Coulter) and in R v4.3.1.

For the functional evaluation of TXNIP in MLR (n=6), cells were labeled to measure T cell proliferation and activation status after 6 days of co-culture. Fc receptor blocking was performed on the total cell suspension using human FcR Blocking reagent (cat no. 130-059-901, Miltenyi). Cell viability was performed using Zombie NIR (cat no. 423106, Biolegend). For cell surface phenotyping, antibodies specific for CD4-BUV496, CD25-BUV395, CTLA-4-APC and PD-1-BV421 were used. Cell suspensions were subsequently stained for intracellular phenotyping: cells were fixed and permeabilized using human FoxP3/Transcription Factor Staining Buffer Set (cat no. 00-5523-00, Thermo Fisher Scientific) according to the manufacturer’s directions and stained with FoxP3-PE (clone: PCH101, eBioscience), TXNIP-FITC (clone: JY2, Novus) and Ki67-BV711 (cat no. 350516, BioLegend). Cells were acquired on CytoFLEX LX (Beckman Coulter). Data visualization and analysis were performed using Kaluza Analysis software v2.1 (Beckman Coulter) and in R (v4.3.1).

For T cell receptor (TCR)-engineered T cells characterization (n=4), cells were labelled after thawing and after TXNIP KO by CRISPR-Cas9 genome editing. Fc receptor blocking was performed on the total cell suspension using human FcR Blocking reagent (cat no. 130-059-901, Miltenyi). Cell viability was performed using Maleimide (cat no. 1408, AAT Bioquest). For cell membrane phenotyping, antibodies specific for CD4-BUV496, CD8-PercP and TCR Vβ13.1-PE were used.

### 2.8 Single cell RNA sequencing

For each sample (n=1), 1.5x10^6^ live cells were collected for scRNA-seq. They were washed once with 0.04% BSA in 1X PBS and centrifuged at 300g for 5 minutes before being processed through 10x Cell Multiplexing Oligo Labeling protocol (10x Genomics, USA) according to the manufacturer’s instructions. ~1,600 cells/µl pooled cell suspensions were prepared with equal number of cells per sample: one for MLR samples, one for non-stimulated T cells samples, and one for anti-CD3 and anti-CD28-stimulated T cells samples. Within each pool, 33,000 cells were used for the 10x Chromium Single-Cell 3’ v3.1 protocol with Feature Barcode (10x Genomics, USA), according to the manufacturer’s instructions. Sequencing was performed on a NovaSeq 6000 sequencer (Illumina, USA) with a sequencing depth around 30,000 reads per cell for gene expression libraries.

### 2.9 Single cell RNA sequencing data analysis

Cell Ranger (v6.0.1, 10x Genomics Inc) was applied for demultiplexing, reads mapping against the GRCh38 human reference genome, and UMI counting. Data analysis was then performed in R (v4.3.1). Seurat package (v4.4.0) was used to generate Seurat objects, selecting genes detected in at least 3 cells. Cells with fewer than 200 genes detected or >15% mitochondrial UMI counts were filtered out. Samples were merged in a unique Seurat object then count data normalization and scaling was performed using Seurat with default parameters. Genes were ranked by descending order of residual variance estimated from the “vst” method implemented in the *FindVariableFeatures* function from Seurat. Excluding immunoglobulin, ribosome-protein-coding, and TCR genes (gene symbol with string pattern “^IGK|^IGH|^IGL|^IGJ|^IGS|^IGD|IGFN1”, “^RP([0-9]+-|[LS])”, and “^TRA|^TRB|^TRG” respectively), the top 2,000 genes were identified as highly variable genes and used for Principal Component Analysis (PCA). Harmony (26) (v0.1.1) was applied for batch effect correction then Uniform Manifold Approximation and Projection (UMAP) and clustering using the Louvain algorithm were performed on the harmony reduction. Non-T cell or -MoDC clusters were removed for further analysis.

Additional R packages used alongside the scRNA-seq analysis are tidyverse (v1.3.2), ggpubr (v0.4.0), gridExtra (v2.3), cowplot (v1.1.1), ComplexHeatmap (27) (v2.12.1) and viridis (v0.6.2).

### 2.10 Differential expression analysis

Differential expression analysis was performed on a subset of scRNA-seq data only containing T cells. The *FindMarkers* function from Seurat (v4.4.0) was used with the *MAST* method, min.pct = 0.1 and logfc.threshold = 0. Genes were considered differentially expressed when exhibiting an absolute log2 fold change value superior to 0.25 and an adjusted p-value inferior to 0.05 (based on Bonferroni correction). Venn diagrams were then generated in R using the ggvenn (v0.1.10) package.

Genes of interest expression was explored in R calculating their average expression using Seurat *AverageExpression* function then heatmaps were generated using the ComplexHeatmap (27) (v2.12.1) package with viridis (v0.6.2) package.

### 2.11 TISCH data analysis

The public Tumor Immune Single-Cell Hub (TISCH) database (28) was used to evaluate TXNIP gene expression in a large panel of cancer types and cell types. Datasets annotations and TXNIP gene expression table were downloaded from TISCH website. Cell types were grouped in three compartments: Immune cells, Malignant cells and Stromal cells. TXNIP gene expression level across cell types and datasets was then plotted in a heatmap generated in R with the ComplexHeatmap (27) (v2.12.1) package.

### 2.12 Pan-cancer T cell atlas analysis

A pan-cancer T cell atlas containing scRNA-seq data from 64 treatment-naïve patients and 15 healthy donors (29) was obtained from zenodo database (10.5281/zenodo.13879752) and re-analyzed with Seurat (v4.4.0). A sub-dataset containing CD4^+^ T cells originating from blood, normal adjacent and tumor samples was generated and UMAP was re-run on the harmony reduction.

TXNIP gene expression was evaluated in CD4^+^ T cells across sample types (tumor, normal adjacent and blood) as well as T cell subtypes. To generate the heatmap, TXNIP average expression was calculated using Seurat *AverageExpression* function. The heatmap was then created using the ComplexHeatmap (27) (v2.12.1) package with viridis (v0.6.2) package.

### 2.13 CRISPR-Cas9 knockout of TXNIP

The Alt-R CRISPR-Cas9 system (IDT; Coralville, Iowa, USA) was used to generate TXNIP knockout (KO) cell pools. Guide RNAs sequences were designed using CRISPOR (http://crispor.tefor.net/ (30), ChopChop (https://chopchop.cbu.uib.no/) (31) and Synthego’s sgRNA design tool (https://www.synthego.com/products/bioinformatics/crispr-design-tool). 3 gRNAs were ordered as Alt-R™ CRISPR-Cas9 sgRNA (sgTXNIP1: 5’-AACGACCCTGAAAAGGTGTA-3’; sgTXNIP2a: 5’-GAGATGGTGATCATGAGACC-3’; sgTXNIP2b: 5’-TCGGCTTTGAGCTTCCTCAG-3’). To design the gRNAs, various parameters were assessed as the targeted localization, the targeted isoforms, MIT and CFD specificity scores, predicted efficiency (32) and off-target number by number of mismatches. sgTXNIP1 targets only TXNIP transcript variant 1 (NM_006472) while sgTXNIP2a and sgTXNIP2b target both TXNIP variants (NM_006472 and NM_001313972).

To perform TXNIP KO, freshly isolated primary human CD4^+^ T cells from 6 healthy donors, or frozen PBT cells isolated from 4 heathy donors and transduced with NY-ESO-1 TCR, were activated and stimulated once using complete RPMI GlutaMAX medium supplemented with 1/100 transact (cat no. 130-111-160, Miltenyi) and 25 ng/mL of IL-2 (cat no. 202-IL, R&D system) for 2 days. Cells were then maintained at a concentration of 1x10^6^ cells/mL under complete RPMI + IL-2 (25 ng/mL) for 5 to 6 days to reach sufficient cell number and viability. On day 5 or 6 of activation, 20x10^6^ T cells were washed twice using 1X PBS, spun down, and resuspended in supplemented P3 buffer using P3 Primary Cell 4D-Nucleofector™ X Kit S (cat no. V4XP-3032, Lonza). TXNIP KO was performed by complexing 150 pmol of Cas 9 Nuclease V3 (cat no. 1081059, IDT) with 450 pmol of either sgTNXIP1 (KO_1), sgTXNIP2a (KO_2a), sgTXNIP2b (KO_2b) or a non-targeting control sgRNA (WT) per donor. According to the manufacturer’s protocol, T cells were mixed with Cas9 RNPs and quickly electroporated using 4D-Nucleofector^®^ (Lonza). Cells were then immediately recovered in fresh prewarmed complete RPMI GlutaMAX medium supplemented with 25 ng/mL of IL-2. After 2 to 5 recovery days, cells were collected for knockout validation by sequencing and western blot, and further used for the functional evaluation of TXNIP in MLR or TCR killing assays.

### 2.14 Sanger sequencing

To validate CRISPR-Cas9 KO of TXNIP, 1x10^6^ of cell pools were collected in duplicate, washed twice in ice-cold 1X PBS and spun down. Dry pellets were frozen at -80°C for at least 24h, until further processing. Cell pellets were thawed and gDNA was extracted using QIAamp DNA Mini Kit (cat no. 69504, Qiagen), followed by PCR amplification with Q5 HotStart High Fidelity (cat no. M0494L, NEB) performed with primers designed around the predicted cut site (Fw1: 5’-GTGCTTGTGGAGATCGGATC-3’ and Rev1: 5’-CTCTAATCAGCTTTCACCCT-3’ for sgTXNIP1; Fw2: 5’-GCAGGGCTTGGCAACTTGCT-3’ and Rev2: 5’-TGAGATGCTTCAATCTAATGCC-3’ for sgTXNIP2a and sgTXNIP2b). Sanger sequencing of the PCR amplicon was performed with BigDye™ Terminator v3.1 Sequencing Kit (cat no. 4337458, Applied Biosystems) following the manufacturer’s instructions using DNA Genetic Analyzer 3500XL. Sequences were analyzed with Thermofisher’s SeqScreener tool (https://apps.thermofisher.com/apps/gea-web/#/setup) to evaluate the knockout efficiency, with a specific focus on frameshift rates.

### 2.15 Western blot analyses

For each sample, cell lysates were prepared from 1x10^6^ cells using cell lysis buffer 10X (cat no. 9803S, Cell Signaling Technology) and protease/phosphatase inhibitor 100X (cat no. 5872S, Cell Signaling Technology). Protein extracts were quantified using Pierce™ BCA Protein Assay Kit (cat no. 23225, ThermoFischer Scientific). Cell extracts were then processed using LDS 4X (cat no. NP007, Invitrogen) and NuPAGE™ Sample Reducing Agent 10X (cat no. 11569166, Invitrogen). 15 µg of protein extracts were separated on NuPAGE™ 4-12% Bis-Tris Gels (cat no. NP0322BOX, Invitrogen), blotted onto nitrocellulose membrane (cat no. 1704158, BioRad) and detected using the following primary antibodies: anti-TXNIP (1:1000)(clone: JY2, MBL) and anti-actin-Rhodamine (1:1000)(cat no. 12004163, BioRad) and the following secondary antibody: anti-mouse IgG1-HRP (1:1000)(cat no.96714S, Cell Signaling). Bands were visualized by Clarity Max™ Western ECL Substrate kit (cat no. 1705062, BioRad) or Rhodamine and quantified by BioRad Image Lab version 6.1.

### 2.16 TCR killing assay

HEK293T and A375 cells were cultured in complete DMEM GlutaMAX culture media consisting of DMEM GlutaMAX (cat no. 61965, Gibco) supplemented with 10% heat inactivated serum (cat no. CVFSVF06-01, Eurobio), 1% P/S (cat no. 15140, Gibco), and 10mM HEPES (cat no. 15630, Gibco). For cell maintenance, adherent cells were washed with PBS (cat no. 14190, Gibco), detached with trypLE Express (cat no. 10043382, Gibco) and plated at 6x10^4^ cells/mL with complete DMEM GlutaMAX culture media twice a week. A375-fluoresecent red cells were obtained by transduction of A375 cells with Incucyte^®^ Nuclight Red Lentivirus (EF1a, Puro) (cat no. 4476, Sartorius) following manufacturer’s instructions. Cells were cultured in complete DMEM GlutaMAX culture media supplemented with 1 µg/mL of puromycin (cat no. A11138-03, Gibco).

For lentivirus production, HEK293T were transfected with 100 µL of TurboFect (cat no. R0534, ThermoFischer) transfection agent, 67.5 µL of the packaging plasmid mix [a 3rd generation packaging system containing 1 mg/mL of pRSV-REV, 1 mg/mL of pMDLg/pRRE and 0.5 mg/mL of pVSV-G] and 15 µg of the transfer lentivector encoding TCRs specific for HLA-A2/NY-ESO1157-165 (33). After 2 days, supernatant containing lentiviruses was collected, centrifuged (1000 g for 5 min at 4°C) and filtered using Steriflip PVDF 0.45 µM (cat no. SE1M003M00, Merck-Millipore). Viral particles were concentrated 7-fold by centrifugation (10000 g for 4 hours at 4°C without brakes) and resuspended in cold complete RMPI GlutaMAX culture media, then snap frozen until further use.

TCR-engineered T cells were generated using PBT cells isolated from 4 heathy donors (D1, D2, D3 and D4) activated for 14 days using complete X-Vivo15 culture medium consisting of X-Vivo15 (cat no. 02-060Q, Lonza) supplemented with 10% inactivated serum Human serum AB (cat no. GEM-100-512-H, BIOIVT), with 1/100 transact (cat no. 130-111-160, Miltenyi) and 25 ng/mL of IL-2 (cat no. 202-IL, R&D system). After 14 days of activation, transduction was performed by adding 1mg/ml of Synperonic F 108 (cat no. 07579-250G-F, SigmaAldrich) with 1 mL of the frozen NY-ESO-1 TCR lentiviruses (TCR condition) or without lentivirus (NT condition) and 25 ng/mL of IL-2 (cat no. 202-IL, R&D Systems). The cells were then maintained under complete X-Vivo15 culture media supplemented with 25 ng/mL of IL-2 for 14 days and cells were frozen until further use. For TXNIP deletion, CRISPR-Cas9 KO TXNIP was performed then TCR, CD4 and CD8 expression were characterized by flow cytometry as described above.

After TXNIP removal from TCR-engineered T cells, TCR T cell killing assay was performed. A375-floresecent red cells were plated at 5000 cells/well in complete DMEM GlutaMAX culture media. The next day, NT (Non transduced), NY WT (NY-ESO-1 TCR-transduced wild type) or NY KO (NY-ESO-1 TCR-transduced and CRISPR-Cas9 KO for TXNIP) engineered T cells from 4 donors (TCR D1, TCR D2, TCR D3, TCR D4) were added in triplicate at different effector to target (E.T) ratios with medium alone or treated with 20µg/mL of anti-PD-1 (nivolumab) or IgG4 isotype control for 13 days. At day 3, 6 and 9, supernatants were removed, and stimulation was performed by adding 5000 cells/well of A375-fluorescent red cells with medium alone or with 20 µg/mL of anti-PD-1 (nivolumab) or IgG4 isotype control. The TCR T cell killing capacity of the A375 tumor cells was assessed by the tumor growth monitored every 8 hours throughout the experiment, with objective 10x and 5 images/well, using the total red nucleus area µm2/well (error bar calculation: Standard Deviation). Data acquisition was performed by Incucyte system (IncuCyte^®^S3, Sartorius) and data visualization were performed using GraphPad Prism 9.0.0 (GraphPad).

## 3 Results

### 3.1 Glucose deprivation potentiates anti-PD-1 immune responses

To evaluate the impact of glucose availability on CD4^+^ T cell responses, we have developed an allogeneic one-way mixed lymphocyte reaction (MLR) assay under high glucose (HG) *versus* low glucose (LG) conditions, with or without anti-PD-1 treatment (**Figure 1A**). Cells and supernatants were harvested after 5 days.

**Figure 2:**
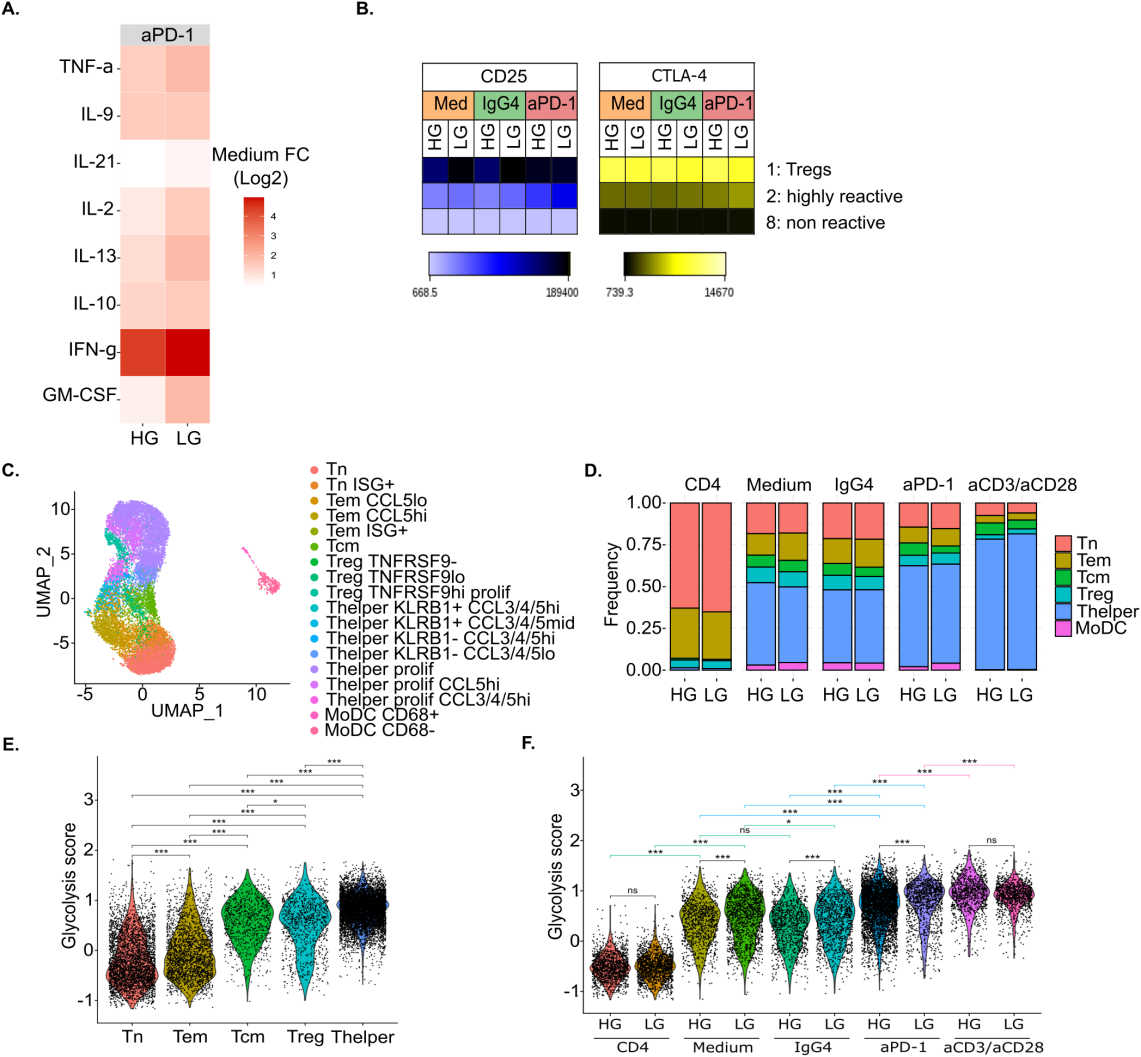
*In vitro* glucose deprivation upon anti-PD-1 stimulation impacts both CD4^+^ T cell immunoreactivity and immunometabolism. **(A)** Heatmap of the log2 fold change (FC) of each cytokine and chemokine concentration in anti-PD-1-treated MLR over untreated MLR (Medium) (n=4). **(B)** Heatmap of the mean fluorescent intensity (MFI) of CD25 and CTLA-4 per conditions and clusters upon MLR stimulation (n=4). **(C)** UMAP representation of *in vitro* scRNA-seq data colored by cell phenotype (n=1). **(D)** Barplot of cell proportions per condition colored by main cell phenotype (n=1). **(E, F)** Violin plot of glycolysis score per main cell phenotype **(E)**, and condition **(F)** (n=1). Statistical analyses for E and F: ANOVA with Tukey’s multiple comparisons tests. *p-value < 0.05, **p-value < 0.01 and ***p-value < 0.001 between indicated groups. HG, High Glucose (11 mM); LG, Low Glucose (1mM).

**Figure 3:**
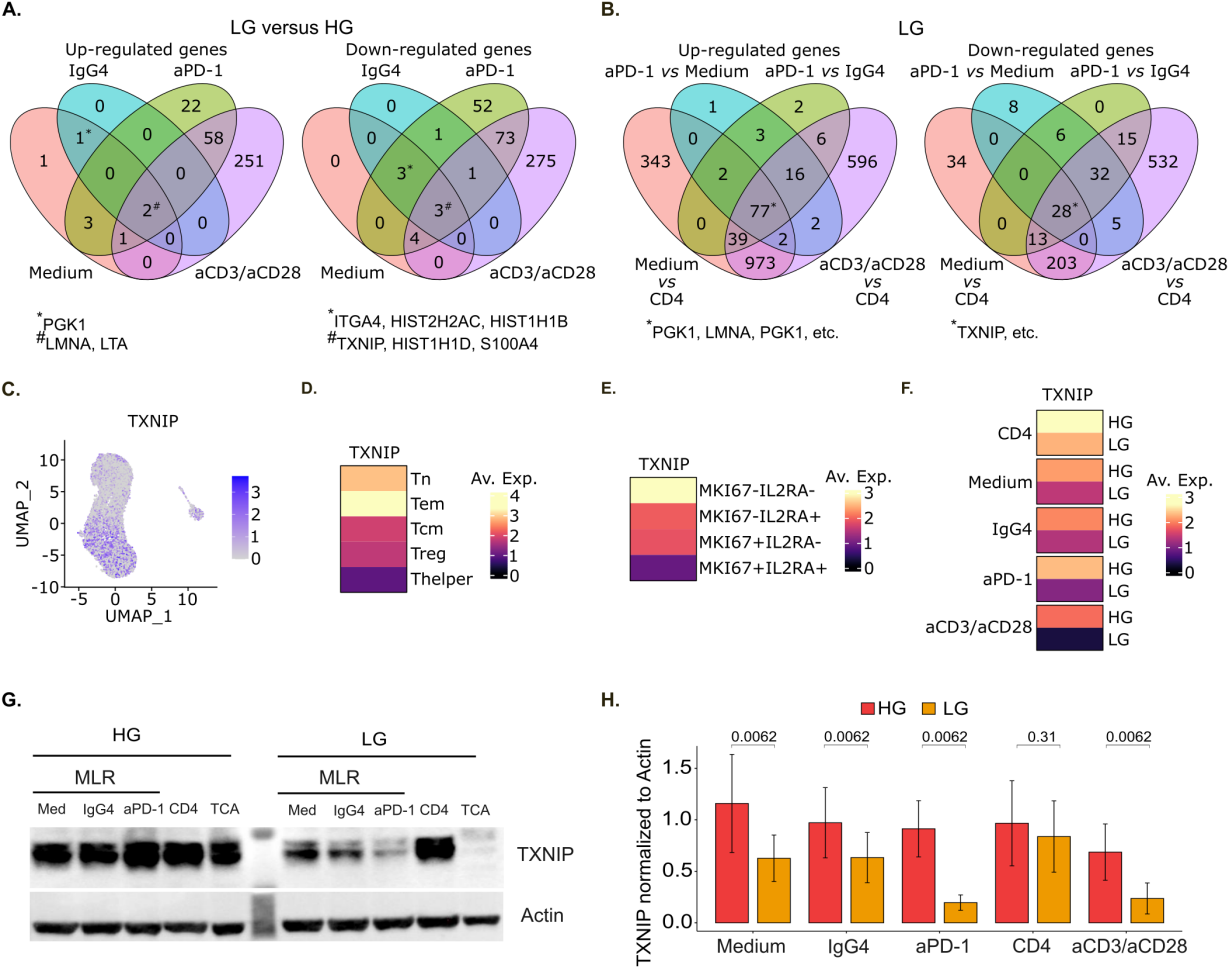
TXNIP is down-regulated both upon CD4^+^ T cell activation and glucose deprivation in MLR. **(A)** Venn diagram of up-regulated (left panel) and down-regulated (right panel) genes in low glucose (LG) compared to high glucose (HG) for the indicated stimulation conditions (n=1). **(B)** Venn diagram of up-regulated (left panel) and down-regulated (right panel) genes in low glucose (LG) for the indicated stimulation conditions comparisons. For A and B, genes exhibiting an absolute log2 fold change value superior to 0.25 and a Bonferroni-adjusted p-value inferior to 0.05 were considered significantly up- or down-regulated (n=1). **(C)** UMAP representation of TXNIP gene expression (n=1). **(D-F)** Heatmap of TXNIP mean gene expression per main cell phenotype **(D)**, MKI67-IL2RA co-expression status **(E)**, and conditions **(F)** (n=1). **(G)** Representative immunoblot of TXNIP and actin expression under high or low glucose concentration. **(H)** Barplot of the corresponding TXNIP expression over actin quantification (n=5). HG, High Glucose (11 mM); LG, Low Glucose (1 mM).

First, we investigated phenotypic changes using multiparametric flow cytometry (18-color panel) and performing UMAP dimensionality reduction and unsupervised clustering on four distinct MLRs. Cells separated into 20 clusters, based on the expression levels of the studied protein markers (**Figure 1B**, **Supplementary Figures S1A, B**). As shown comparing the controls (CD4 non-stimulated T cells and anti-CD3 and anti-CD28 stimulation), MLRs worked as expected and induced T cell immune responses similar to the ones described previously (34). Interestingly, we noticed some differences in T cell responsiveness between HG and LG. Upon glucose deprivation, immunoreactive cluster 2, which represents effector T cells, was slightly reduced (Medium: 64.3% +/- 6.4; aPD-1: 71.2% +/- 5.8) compared to HG (Medium: 66.7% +/- 6.7; aPD-1: 78.3% +/- 4.9) whereas non-reactive cluster 8 was moderately increased (Medium: 14.8% +/- 4.9; aPD-1: 12.6% +/- 6.9) compared to HG (Medium: 9.1% +/- 7.4; aPD-1: 6.5% +/- 6.2) in both untreated MLR (Medium) and MLR treated with anti-PD-1 (aPD-1); nonetheless we also observed that cells in LG conditions remained anti-PD-1 reactive (**Figures 1B, C**, **Supplementary Figure S1B**). To assess the effects of glucose deprivation on CD4^+^ T cell effector functions, we evaluated cytokine secretion and immune checkpoints (ICPs) expression upon anti-PD-1 stimulation. Cytokine production was evaluated by a multiplex cytokine assay on the four MLRs. We compared the fold change of anti-PD-1-treated MLR over untreated MLR (Medium) or MLR treated with isotype control (IgG4) in HG *versus* LG conditions. Most of the tested soluble immune factors showed an increase in concentration after anti-PD-1 treatment (log2FC > 0.5) compared to untreated MLR (**Figure 2A**, **Supplementary Figures S2A, B**). Surprisingly, the fold change also increased further under glucose deprivation, with IFN-γ being the predominant released cytokine (log2FC = 4.96 in LG *versus* log2FC = 4.37 in HG) (**Figure 3A**, **Supplementary Figures S2A, B**). We also examined ICPs expression by flow cytometry in immunoreactive cells and observed that, specifically in highly reactive cluster 2, CD25 and CTLA-4 were the only tested markers that appeared to be modulated by low glucose. CD25 and CTLA-4 expression were slightly increased in the anti-PD-1 treated LG (CD25 MFI = 77762 and CTLA-4 MFI = 4909) condition compared to HG (CD25 MFI = 49686 and CTLA-4 MFI = 4215) (**Figure 2B**).

We further characterized immune cells in HG *versus* LG conditions by performing scRNA-seq on one donor. We obtained transcriptomic data for 15,577 cells. Following integration, cells separated into 18 clusters that could be assigned to T cells and MoDCs subtypes based on both differential gene expression and interrogation of known gene markers expression (**Figure 2C**, **Supplementary Figure S3**) (35–37). In total, we identified 2 subtypes of MoDCs that mainly separated on CD68 expression, and 16 subtypes of CD4^+^ T cells including naïve T cells (Tn), effector memory T cells (Tem), central memory T cells (Tcm), regulatory T cells (Treg), and T helper cells (Thelper or effector T cells).

Cell composition analysis showed a large increase in Thelper from around 47% and 43% respectively in untreated MLR (Medium) and MLR treated with isotype control (IgG4) to 60% upon anti-PD-1 treatment while the Treg fraction slightly decreased from 9% to 6% in the same conditions (**Figure 2D**). However, we did not observe here a clear impact of glucose deprivation on cell proportions, irrespective of T cell stimulation.

In accordance with multiplex cytokine assay data, we could observe that, upon anti-PD-1 treatment, the percentage of IFNG-expressing cells increased by 13.2% under LG (22.0% of IFNG-expressing CD4^+^ T cells under anti-PD-1 treatment *versus* 8.8% in untreated MLR) while only 5.9% increased under HG (13.1% *versus* 7.2%) (**Supplementary Figure S4**). Similarly, the percentage of CTLA-4-expressing cells increased by 8.5% under LG (51.5% *versus* 43.0%) while only 2% increased under HG (46.5 *versus* 44.5%) upon anti-PD-1 treatment. Moreover, scRNA-seq allowed us to explore more markers of CD4^+^ T cells activation. Among them, we found that, likewise CTLA4, PDCD1 gene expression increased upon anti-PD-1 treatment by 7.9% under glucose deprivation (23.7% *versus* 15.8%).

We then explored the metabolic adaptation of immune cells to glucose deprivation at the transcriptomic level. For each single cell, we proposed a glycolysis score computed with the *AddModuleScore* function from Seurat package and based on the expression of ENO1, GAPDH, GPI, PGAM1, PGK1, PKM and TPI1 genes that encode for enzymes of the glycolysis pathway (38). We observed that the glycolysis score was significantly higher in activated Tcm, Treg and Thelper cells compared to naïve T cells (Tn) and effector memory T cells (Tem) (**Figure 2E**). Moreover, the glycolysis score was higher in MLR-stimulated compared to non-stimulated T cells and it further increased in anti-PD-1-treated MLR and even more upon anti-CD3 and anti-CD28 stimulation (**Figure 2F**). This indicates that activated T cells feature enhanced transcriptomic glycolytic metabolism. Surprisingly, the glycolysis score was significantly higher under low glucose compared to high glucose for all MLR-stimulated conditions. This suggests that glucose-deprived T cells undergo adaptations to support their enhanced metabolic needs upon activation.

Therefore, in this MLR setting, glucose deprivation seems to potentiate anti-PD-1 immune responses of CD4^+^ T cells through enhanced glycolytic metabolism leading to increased IFN-γ secretion as well as CTLA-4 and PD-1 expression.

### 3.2 TXNIP links glucose metabolism and T cell activation

With the aim of identifying key molecular drivers of T cell activation under glucose deprivation, we performed a differential expression analysis on scRNA-seq data comparing LG and HG conditions across stimulations. Only 3 genes, namely PGK1, LMNA and LTA, were significantly up-regulated in LG *versus* HG upon untreated condition and MLR treated with isotype control (**Figure 3A**, **Supplementary Table S1**). The number of significantly up-regulated genes then reached 86 and 312 upon MLR treated with anti-PD-1 or anti-CD3 and anti-CD28 stimulation, respectively, both including LMNA and LTA genes. As for down-regulated genes, 6 were overlapping in LG *versus* HG upon untreated, isotype control-treated and anti-PD-1-treated MLR stimulations: TXNIP, HIST1H1D, ITGA4, S100A4, HIST2H2AC and HIST1H1B (**Figure 3A**, **Supplementary Table S1**). Among them, TXNIP, HIST1H1D and S100A4 were also down-regulated in LG *versus* HG upon anti-CD3 and anti-CD28 stimulation.

We also performed differential expression analysis to study the effect of different stimulations under glucose deprivation. Interestingly, PGK1, LMNA and LTA were among the 77 significantly up-regulated genes upon all the different stimulations, while TXNIP was among the 28 down-regulated genes (**Figure 3B**, **Supplementary Table S1**) across all conditions.

In short, the two sets of differential expression analyses showed that PGK1, LMNA and LTA genes are up-regulated while TXNIP is down-regulated both upon CD4^+^ T cell activation and glucose deprivation. PGK1 gene encodes for the phosphoglycerate kinase 1 protein which is a glycolytic enzyme catalyzing the conversion of 1,3-diphosphoglycerate to 3-phosphoglycerate. It was previously shown that PGK1 inhibition with NG52 treatment attenuates autoimmune myocarditis in mice through suppressing CD4^+^ T cell activation and differentiation into Th17 cells (39). The lamins A and C proteins encoded by LMNA gene are part of the nuclear lamina matrix. As such, lamins regulate multiple cellular functions, including higher-order genome organization, DNA replication and repair, gene transcription and signal transduction. It was shown that A-type lamins expression increases rapidly upon T cell receptor activation and that this early event accelerates the formation of the immunological synapse between T cells and antigen-presenting cells (40). LTA gene encodes for the lymphotoxin alpha protein, a cytokine belonging to the tumor necrosis factor family. It is largely described as a mediator of effector immune responses (41). As for TXNIP gene, it encodes for the thioredoxin-interacting protein which is commonly considered an endogenous antagonist of thioredoxin (TRX), a key regulator in cellular redox balance (7, 8). In T cells, it was shown to negatively regulate glucose uptake as well as cell proliferation (19, 24). However, TXNIP impact on T cell effector functions remains poorly understood. Thus, we decided to further characterize TXNIP immunoregulatory role in T cells.

We checked TXNIP gene expression levels in the studied *in vitro* conditions and showed that TXNIP was mainly expressed by Tn and Tem (**Figures 3C, D**). Its expression level then decreased in other activated T cells subtypes, and particularly in cells expressing MKI67 and IL2RA (**Figures 3D, E**). In line with this, under glucose deprivation, TXNIP expression level was lower in MLR-stimulated compared to non-stimulated T cells and it further decreased in anti-PD-1-treated MLR and even more upon anti-CD3 and anti-CD28 stimulation (**Figure 3F**). Additionally, TXNIP expression level was lower under LG *versus* HG for all stimulation conditions (**Figure 3F**). We also analyzed the corresponding regulation of TXNIP at the protein level by western blot in five other sets of MLR (**Figures 3G, H**). We confirmed that TXNIP protein expression significantly decreased at low glucose concentration in untreated MLR or treated with isotype control (IgG4), and even more drastically in MLR treated with anti-PD-1 or after single stimulation with anti-CD3 and anti-CD28 antibodies. Hence, we showed for the first time in human CD4^+^ T cells that TXNIP is down-regulated both upon T cell activation and glucose deprivation.

### 3.3 TXNIP is highly expressed in lymphocytes and its expression level decreases in activated tumor-infiltrating CD4^+^ T cells

**Figure 4:**
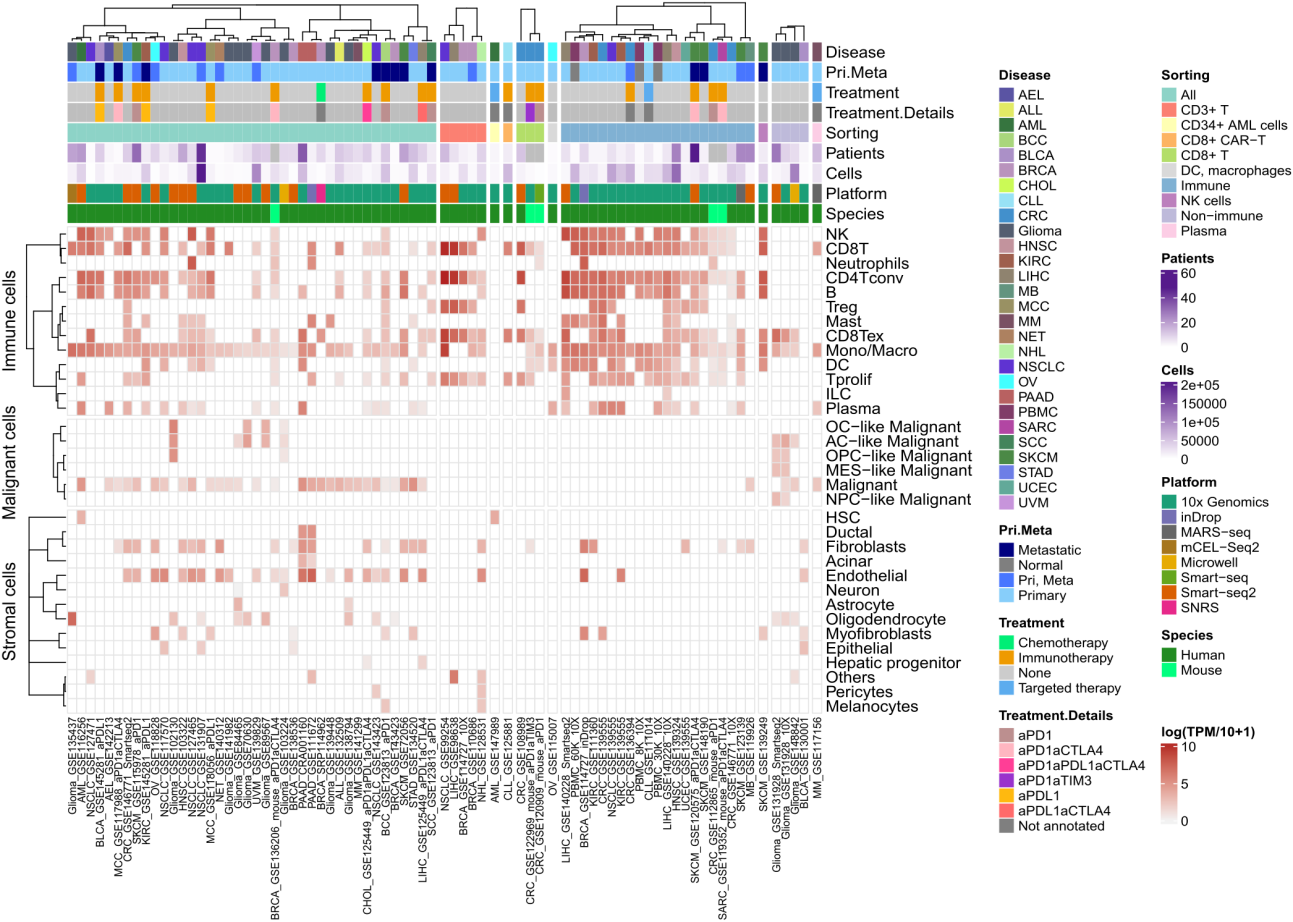
In patients TXNIP is highly expressed in lymphocytes. Heatmap of TXNIP mean gene expression computed as log(TPM/10 + 1) across nearly 2 million single cells in total, per cell phenotype (as rows) and dataset (as columns) in TISCH database. All metadata available in TISCH for each dataset was included in annotation on top of the heatmap.

To evaluate the translatability of these *in vitro* findings to the clinic, we then explored TXNIP gene expression in cancer patients’ data. We explored TXNIP gene expression at the single cell level using TISCH database (28). Across all scRNA-seq datasets available in TISCH (*i.e.* 78 datasets across 28 cancer types), TXNIP showed high expression levels in immune cells, fibroblasts and endothelial cells as well as malignant cells, irrespective of the cancer type (**Figure 4**). Importantly, its expression level was slightly higher in lymphocytes compared to other cell types in each dataset. Hence, TXNIP is highly expressed in lymphocytes but not specific to this cell population.

**Figure 5:**
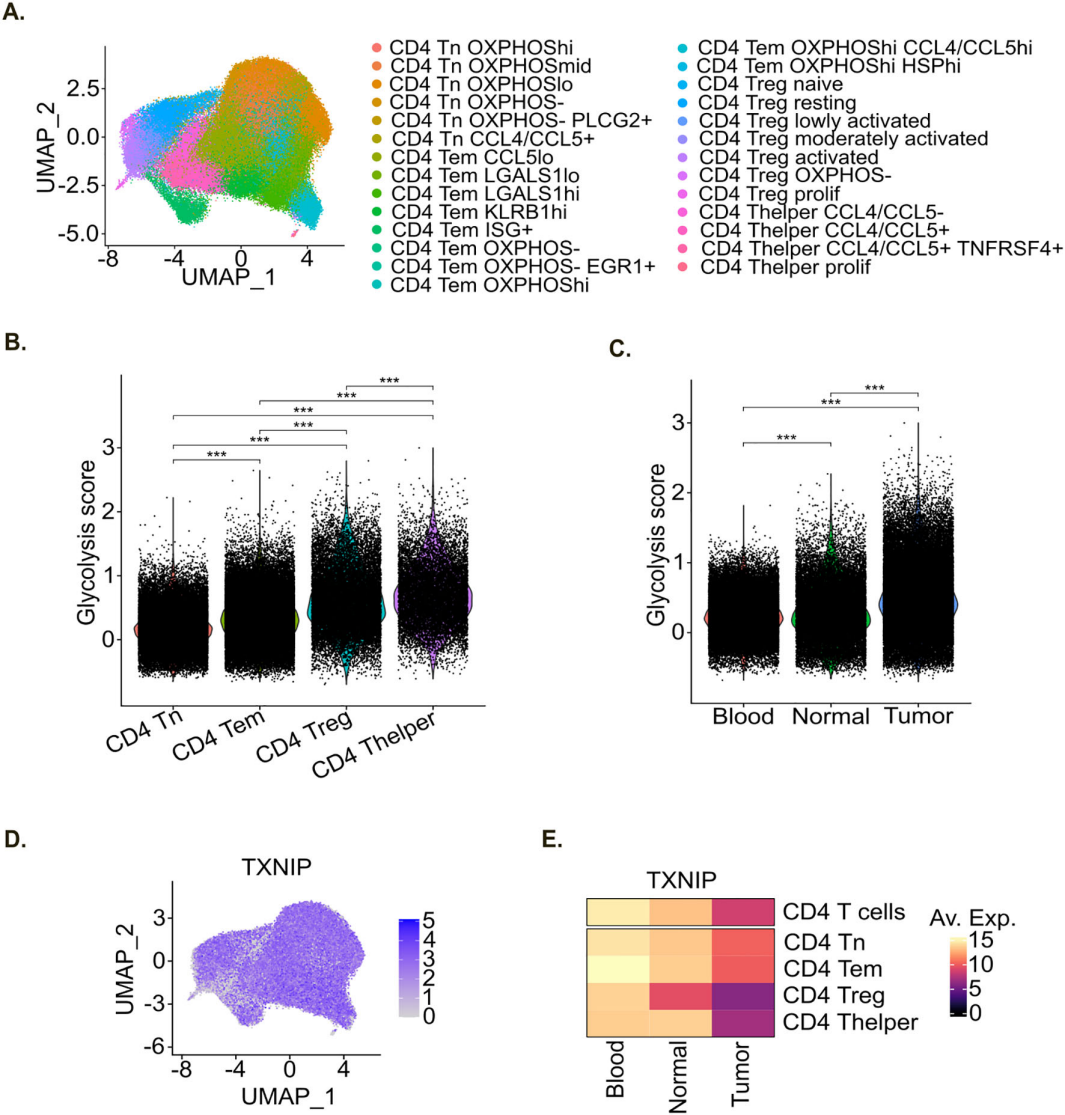
In patients TXNIP expression level decreases in tumor-infiltrating CD4+ T cells. **(A)** UMAP representation of the pan-cancer scRNA-seq CD4^+^ T cell atlas colored by cell phenotype. **(B, C)** Violin plot of glycolysis score within the pan-cancer scRNA-seq CD4^+^ T cell atlas per main cell phenotype **(B)**, and sample type **(C)**. Statistical analyses: ANOVA with Tukey’s multiple comparisons tests. *p-value < 0.05, **p-value < 0.01 and ***p-value < 0.001 between indicated groups. **(D)** UMAP representation of TXNIP RNA expression within the pan-cancer scRNA-seq CD4^+^ T cell atlas. **(E)** Heatmap of TXNIP mean gene expression per sample type within the pan-cancer scRNA-seq CD4^+^ T cell atlas.

To further evaluate the translatability of our *in vitro* findings to patients, we re-analyzed a scRNA-seq pan-cancer T cell atlas (29). We generated from this atlas a CD4^+^ T cell sub-atlas comprising scRNA-seq data for 119,960 CD4^+^ T cells from 64 treatment-naïve patients and 10 healthy donors, including colorectal cancer (CRC) (42), intrahepatic cholangiocarcinoma (CHOL) and hepatocellular carcinoma (HCC) (43), head and neck squamous cell carcinoma (HNSCC) (44), non-small cell lung cancer (NSCLC) (42), pancreatic ductal adenocarcinoma (PDAC) (45), renal cell carcinoma (RCC) (42, 46) and uterine corpus endometrial carcinoma (UCEC) (42) (**Supplementary Figure S5A**). CD4^+^ T cells in this new scRNA-seq sub-atlas originated from tumor, adjacent normal tissue and blood samples (**Supplementary Figure S5B**). As data integration was run again on the CD4^+^ T cell dataset after subsetting from the pan-cancer T cell atlas (34), we checked its quality by dimension reduction. This showed good data integration with cells not clustering by dataset of origin (**Supplementary Figure S5A**) or sample type (**Supplementary Figure S5B**), but by cell annotation which was recovered from the pan-cancer T cell atlas (**Figure 5A**) (29).

Likewise *in vitro* analysis, we explored the glucose metabolism of CD4^+^ T cells in this atlas by computing a glycolysis score for each single cell. Similarly to *in vitro* results (**Figure 2E**), we observed that the glycolysis score was significantly higher in activated Treg and Thelper cells compared to Tn and Tem cells in patients (**Figure 5B**). Moreover, the glycolysis score was higher in CD4^+^ T cells originating from adjacent normal tissue compared to blood and it further increased in CD4^+^ T cells originating from the tumor (**Figure 5C**).

We then evaluated TXNIP gene expression in this CD4^+^ T cell atlas. TXNIP gene was expressed broadly and at high levels (**Figure 5D**). Its expression level was globally higher in Tn and Tem compared to activated Treg and Thelper populations (**Figure 5E**) which is in line with our *in vitro* data (**Figure 3D**). Moreover, TXNIP gene expression level was higher in CD4^+^ T cells originating from blood compared to adjacent normal tissue, and even more compared to tumor samples (**Figure 5E**). This decrease in TXNIP gene expression level in tumor was also observed within each CD4^+^ T cell subtype, indicating that this decrease not only results from changes in main cell subtypes composition but also from intrinsic transcriptomic adaptation to tumor micro-environment.

### 3.4 TXNIP deletion in T cells enhances their effector functions

**Figure 6:**
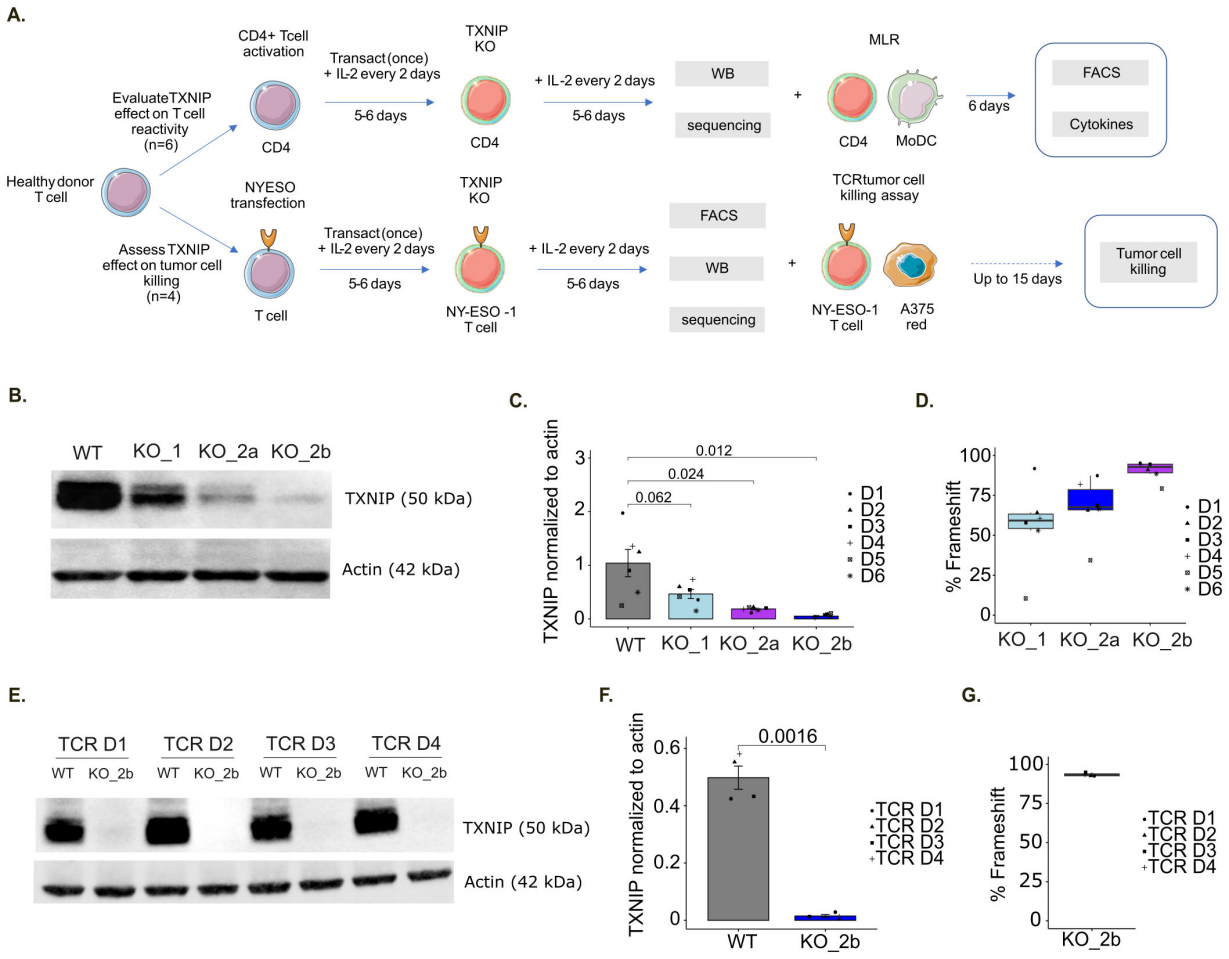
TXNIP knockout in immune T cells. **(A)** Schematic representation of the *in vitro* assay protocol for TXNIP functional evaluation. **(B)** Representative immunoblot of TXNIP and actin expression upon TXNIP-targeting sgRNA in MLR donors: WT (scramble sgRNA), KO_1 (sgTXNIP1), KO_2a (sgTXNIP2a), KO_2b (sgTXNIP2b). **(C)** Barplot of the corresponding TXNIP expression over actin quantification (n=6). **(D)** Box plot of predicted percentage of frameshift for the indicated TXNIP-targeting sgRNA compared to WT determined by Sanger sequencing for MLR samples (n=6). **(E)** Representative immunoblot of TXNIP and actin expression upon TXNIP-targeting sgRNA in TCR donors: WT (scramble sgRNA), KO_2b (sgTXNIP2b). **(F)** Barplot of the corresponding TXNIP expression over actin quantification (n=4). **(G)** Box plot of predicted percentage of frameshift for KO_2b compared to WT determined by Sanger sequencing for TCR killing assay samples (n=4).

To evaluate the impact of TXNIP on T cell activation and immunoreactivity, we performed a TXNIP-targeted knockout (KO) and examined resulting T cell functions. We designed three guides targeting either the longest TXNIP transcript (ENST00000582401) with sgTXNIP1 or both protein coding transcripts (ENST00000582401 and ENST00000425134) with sgTXNIP2a and sgTXNIP2b (**Supplementary Figure S5C**), resulting in the generation of 3 different KO (KO_1, KO_2a and KO_2b, respectively). We then performed TXNIP KO on freshly isolated CD4^+^ T cells from six healthy donors (**Figure 6A**). TXNIP deletion was validated by measuring the corresponding protein target expression (**Figures 6B, C**) and genomic frameshift rates (**Figure 6D**) in comparison with a non-targeting control guide (scramble sgRNA resulting in WT CD4^+^ T cells). Of note, KO_2b (sgTXNIP2b) was found to be the most efficient guide with a frameshift rate above 90% inducing near-total deletion of TXNIP protein expression across the six donors. Based on these data, we assessed the functional activity of T cells using KO_2b.

**Figure 7:**
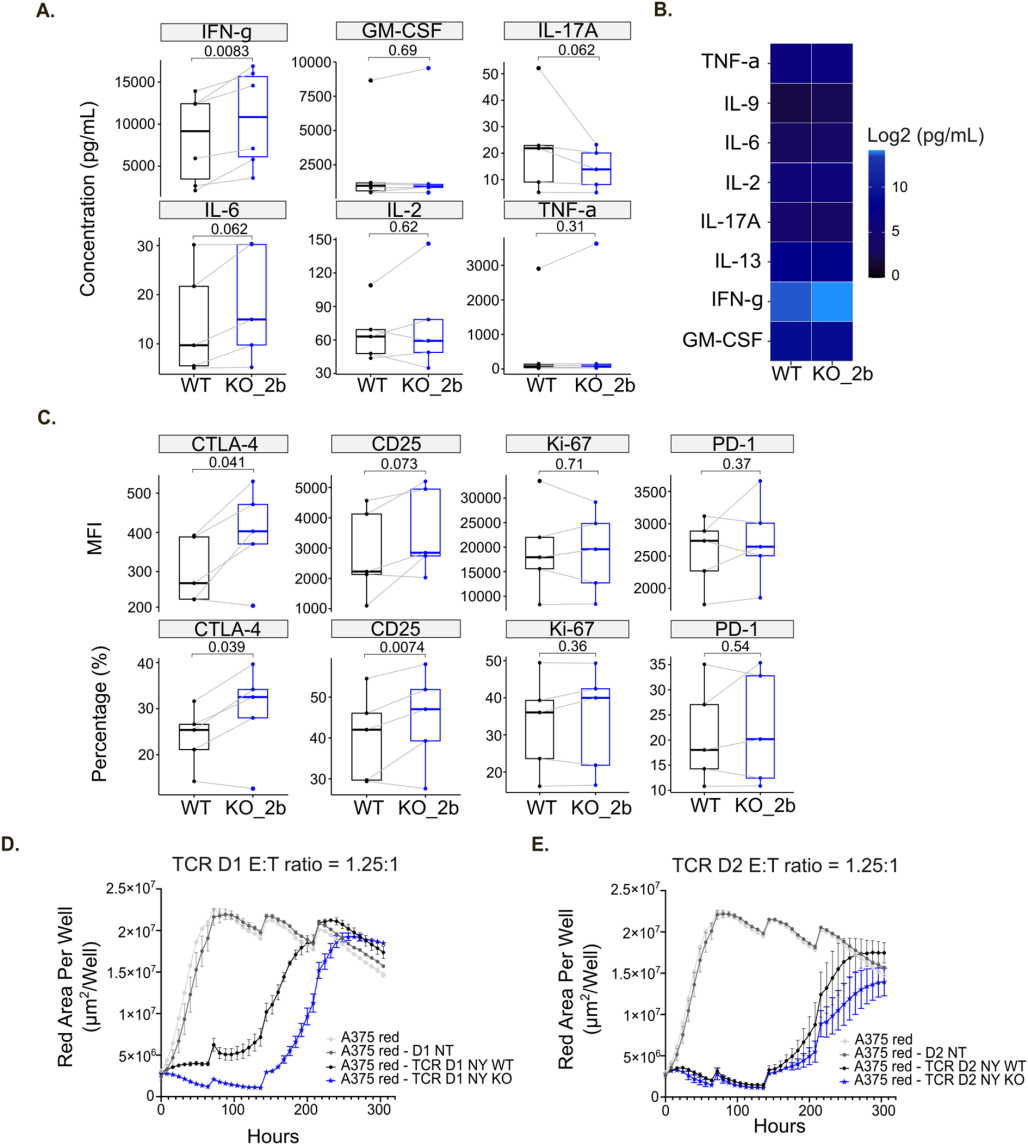
TXNIP knockout increases T cell immune responses. **(A)** Box plots of indicated soluble factor concentrations in untreated MLR (Medium), displaying group of numerical data through their 3^rd^ and 1^st^ quantiles (box), median (central band), minimum and maximum (whiskers) (n=6). Statistical analyses: Wilcoxon or T test, p-value is considered significantly relevant when p< 0.05 for the corresponding soluble factor. **(B)** Heatmap of the log2 concentration of the indicated cytokines and chemokines in untreated MLR (Medium) (n=6). **(C)** Box plots of the mean fluorescent intensity (MFI) or percentage (%) for the indicated markers in untreated MLR (Medium), displaying group of numerical data through their 3^rd^ and 1^st^ quantiles (box), median (central band), minimum and maximum (whiskers) (n=5). Statistical analyses: Wilcoxon or T test, p-value is considered significantly relevant when p<0.05 for the corresponding soluble factor. **(D, E)** Red Area Per Well of NY-ESO-1^+^ A375 tumor cells was plotted over time alone or after addition of NT (non-transduced), NY WT (TCR only) and NY KO (TCR with TXNIP KO) T cells for donor 1 (D1) **(D)** or donor 2 (D2) **(E)**.

Following validation of TXNIP depletion, we performed a MLR assay using TXNIP KO CD4^+^ T cells (**Figure 6A**). On day 6, we evaluated CD4^+^ T cell reactivity by measuring cytokine release (**Figures 7A, B**) together with T cell proliferation and activation status by flow cytometry (**Figure 7C**). Among the soluble immune factors included in the panel, IFN-γ showed a significant increase in TXNIP KO (KO_2b) condition compared with the non-targeting KO control (WT) (*p=0.0083*). To further assess the immune response in this setting, we examined the expression of CTLA-4, PD-1, CD25 and Ki-67 by flow cytometry. We observed that CTLA-4 expression was significantly higher in TXNIP-deficient CD4^+^ T cells compared to WT control CD4^+^ T cells (CTLA-4 MFI, *p=0.041*; CTLA-4 percentage, *p=0.039*). T cell activation is slightly increased by TXNIP KO (CD25 MFI, *p=0.073*; CD25 percentage, *p=0.073*), while Ki-67 and PD-1 expression is not affected (**Figure 7C**). Of note, anti-PD-1 usually further increases activation of T cells, but we do not observe this in our settings (**Supplementary Figures S6A, B**). Overall, IFN-γ secretion, as well as CTLA-4 and CD25 expression, highlighted the enhanced immune responses of T cells lacking TXNIP expression in an MLR setting.

To further validate the functional activity of TXNIP removal in primary human T cells, we assessed the effect of TXNIP KO in T cells on tumor growth *in vitro* by performing an antigen specific T cell killing assay (**Figure 6A**). Briefly, T cells isolated from four primary human PBMCs were transduced (TCR) or not (NT) with the previously described NY-ESO-1 TCR system (33, 47). TXNIP KO was then performed on NY-ESO-1-TCR cells. Again, the efficiency of TXNIP KO was evaluated by western blot and sequencing. The western blot confirmed the significant loss of TXNIP protein expression, and the frameshift rate highlighted efficient deletion of the gene in all four donors (**Figures 6E–G**). TXNIP depletion did not affect cell proportion or percentage of NY-ESO-1-TCR expression on T cells (**Supplementary Figure S6C, D**). Subsequently, T cell killing in response to HLA-A2^+^ A375 tumor cells presenting NY-ESO-1 was compared between NT (non-transduced), NY WT (TCR only) and NY KO (TCR with TXNIP KO) T cells. For all 4 donors, NT T cells had no impact on killing kinetics and NY WT T cells effectively provided potent tumor growth control. For donors 1 and 2, NY KO T cells elicited higher cytotoxicity against NY-ESO-1^+^ A375 tumor cells compared to NY WT T cells (**Figures 7D, E**). Of note, donors 3 and 4 did not exhibited significant differences in the rate of killing when TXNIP was depleted. These differences in TXNIP KO effect on T cell tumor control correlated with anti-PD-1 responsiveness status, donors 1 and 2 being anti-PD-1 responsive while donors 3 and 4 were not (**Supplementary Figures S6E, F**). Hence, we observed that, in a T cell killing assay setting, TXNIP depletion seems to have the potential to enhance the cytotoxic activity of effector T cells.

Taken together, these data highlight the role of TXNIP in T cell immune responses and tumor cell killing capacity.

## 4 Discussion

Metabolic reprogramming is an important hallmark of cancer initiation and progression (48). Cancer cells require distinct and diversified cellular metabolisms to follow high proliferative rates, meet biomass production needs and to adapt to micro-environmental changes. Metabolic adaptation together with exchange of metabolites and growth factors between cells also regulate gene and protein expression (49). Import of more nutrients, coming from conventional and non-conventional sources, and refocus of their metabolic pathways allow them to establish ceaseless pro-tumoral signals (49, 50). Among them, tumor cells mostly reprogram their metabolism by high glucose consumption and aerobic glycolysis (*i.e.*, the Warburg effect) to support their growth and spreading (1, 2). As a result, the TME is highly complex with specific and drastic conditions, such as nutrient starvation. T cells present in the TME compete with tumor cells for nutrients availability, like glucose, as they also need nutrients to survive, to get activated and to proliferate efficiently. Studies have demonstrated that these biochemical specificities impact immune cells functions, reducing antitumor activity (3). To evaluate in more details the effect of glucose deprivation on T cell immune reactivity, we developed a MLR assay at the glucose level similar to those found in the TME (*i.e.* 1 mM), compared to conventional glucose concentration used in culture conditions (*i.e.* 11 mM).

As expected, the low glucose condition lowered T cell immunoreactivity. Nevertheless, even though glucose restriction impacted T cell reactivity, immune cells remained responsive to PD-1 blockade. In our settings, we observed that low glucose induced higher IFN-γ secretion as well as CD25 and CTLA-4 expression under anti-PD-1 stimulation, indicating that glucose restriction may enhance anti-PD-1 immune responses. The evaluation of glycolysis-related genes by scRNA-seq showed a potential increased activation of this pathway upon MLR or anti-CD3 and anti-CD28 stimulation. The expression of these glycolysis-related genes was higher under low glucose compared to high glucose for all MLR conditions. This suggests that glucose-deprived T cells undergo metabolic adaptation, boosting their glycolysis to meet their energy requirements upon activation.

Aerobic glycolysis enhances IFN-γ production (51) and it was previously shown that an increase in GLUT-1 and glycolysis after transient glucose deprivation in CD8^+^ T cells resulted in enhanced anti-tumor functions *in-vivo* (52, 53). Yet, the authors did not assess the link between low glucose, increased GLUT-1 and glycolysis. In the present study, using scRNA-seq, we showed that TXNIP, a negative regulator of glucose metabolism, is repressed upon T cell activation and stimulation. TXNIP expression was significantly downregulated both in low glucose compared to high glucose and upon T cell activation. TXNIP is part of the intracellular redox system through its interaction with thioredoxin (TRX) (7, 8), but it also plays a role in the adaptation of energy metabolism via a feedback loop (54). When the intracellular glucose concentration increases, TXNIP is expressed and inhibits glucose uptake through direct GLUT-1 endocytosis and internalization leading to glycolysis reduction (55, 56). In glucose-restricted conditions, TXNIP level is thus reduced, and glycolysis is enhanced. We can hypothesize here that the decrease in TXNIP expression in T cells might probably increase GLUT-1 expression at the cell membrane leading to enhanced glucose consumption. Increased intracellular glucose concentration, together with anti-PD-1 stimulation, can thus lead to enhanced glycolytic metabolism resulting in increased T cell activation and functions.

TXNIP is implicated in multiple intracellular mechanisms (16, 57–59). In immune cells, TXNIP deletion in mice has been shown to increase germinal center B-cell responses, sensitivity to activation and IFN-γ secretion by NK cells (18–20). In T cells, other models have confirmed that TXNIP inhibition modulated IFNG transcription and T cell proliferation upon stimulation (19, 21–23). One study in human T cells has determined that TNF-α induced a down-regulation of TXNIP and this diminution was associated with increased glucose uptake (24). To elucidate the role of TXNIP on immune reactivity and anti-tumor effector functions of T cells, we generated TXNIP-deficient T cells by CRISPR Cas-9 genome editing. Our data showed for the first time in primary human T cells that deletion of TXNIP led to increased IFN-γ production and expression of T cell activation markers (*i.e.* CTLA-4 and CD25) and could result in greater tumor cell killing *in vitro*. These results suggest that specific inhibition of TXNIP could be sufficient to enhance T cell effector functions.

We additionally explored TXNIP expression in cancer patients. scRNA-seq data exhibited that TXNIP is not only expressed in lymphocytes but also in other immune cells, tumor cells and stromal cells, the expression in lymphocytes being the highest. TXNIP expression in tumor cells is well documented but its role remains controversial (16, 60). Initially described as a tumor-suppressor gene, some studies showed a pro-tumoral effect of TXNIP expression. Our analysis highlighted that, in lymphocytes, TXNIP expression was reduced in activated T cells as well as in tumor compared to paired blood and normal tissues. This is in line with our *in vitro* data generated based on a glucose-deprived MLR model. Altogether, studying single cell transcriptomic data generated from patients’ tumor, we observed that TXNIP could have a potential role in T cell activation within the TME.

Notwithstanding the above results, the mechanisms underlying these immune responses remain to be determined. We can hypothesize that TXNIP may have a direct impact on T cell survival, immunometabolism and/or TCR signaling (**Supplementary Figure S7**). Indeed, TXNIP is involved in the regulation of the redox system, its removal can leverage antioxidative activity of TRX and decrease ROS-induced apoptosis (or activation-induced cell death, AICD), thereby increasing T cell survival (61, 62). Furthermore, as glycolytic metabolism is essential for T cell activation, TXNIP depletion can enhance glycolysis, leading to an increase in T cell activation and functions, suggested by the link between TXNIP and the phosphoinositide 3-kinase/protein kinase B (PI3K/Akt) pathway resulting in cell activation and proliferation. In other cell types (murin hepatocytes or tumor cells) PI3K/Akt signaling has been shown to directly inhibit TXNIP. Akt is required to increase GLUT-1 membrane expression through phosphorylation and degradation of TXNIP. Enhanced GLUT-1 expression in turn stimulates glucose uptake and cell proliferation (11, 12, 63, 64). In other systems (insulin models, fasting or diabetic mice), TXNIP indirectly inhibits Akt activity and decreases glycolysis (65–67). TXNIP is an indirect inhibitor of the PI3K/Akt pathway as TXNIP binds to TRX making it no longer available to inactivate PTEN, a PI3K/Akt negative regulator downstream of PD-1. The mechanisms in T cells remain unidentified and further analyses are required to confirm these hypotheses, but as PI3K and Akt are part of CD28 and TCR signaling pathways, we can hypothesize that these phenomena also occur in T cells and are responsible for the observed increase in effector immune functions. TXNIP could act on the CD28 and/or the TCR signaling pathways via indirect activation of PTEN. Down-regulation of TXNIP in T cells should thus increase PI3K/Akt signaling pathway downstream of the TCR and/or CD28, leading to improved glucose immunometabolism and T cell effector functions. TXNIP could therefore represent a link between glycolysis and CD28/TCR signaling.

Altogether, we identified TXNIP as an immunometabolic regulator of T cell activation, highlighting the complexity and great potential of immunometabolism for the development of future therapies against cancer. In particular, TXNIP depletion could be of interest in combination with immunotherapies (such as anti-PD-1 or other immune checkpoint blockers) to boost T cell immunity and/or for the development of next-generation CAR-T cells by improving their anti-tumor efficacy.

## Data Availability

The datasets presented in this study can be found in online repositories. The names of the repository/repositories and accession number(s) can be found below: https://zenodo.org/records/13904077, 10.5281/zenodo.13904077.

## Supplementary material

Supplementary Figure 1
**(A)** Concatenated flow cytometry UMAP dimensionality reduction representation of CD4^+^ T cells under high glucose (HG) or low glucose (LG) colored by indicated markers’ mean fluorescent intensity for all donors (n=4). **(B)** Individual flow cytometry UMAP dimensionality reduction representation of CD4^+^ T cells upon MLR stimulation colored by non-supervised clustering (Donor 2, Donor 3 and Donor 4). HG, High Glucose (11 mM); LG, Low Glucose (1mM).

Supplementary Figure 2
**(A)** Box plots of the Log2 fold change (FC) of each cytokine and chemokine concentration in anti-PD-1-treated MLR over untreated MLR (Medium), displaying group of numerical data through their 3^rd^ and 1^st^ quantiles (box), median (central band), minimum and maximum (whiskers) (n=4). Statistical analyses: p-value is considered significantly relevant when p<0.05 for the corresponding soluble factor. **(B)** Barplot of indicated soluble factor concentrations in untreated (Med), isotype control (IgG4), anti-PD-1-treated (aPD-1) MLR or CD4^+^ T cells alone (CD4) under high glucose (HG, 11 mM) or low glucose (LG, 1 mM) conditions.

Supplementary Figure 3 Bubble plot showing expression of representative signature genes of the *in vitro* scRNA-seq data. Color represents the normalized expression level and size represents the expression frequency (n=1).

Supplementary Figure 4 UMAP representation of RNA expression and box plot of differential percentage of expressing cells for the indicated genes. Delta represents the difference between the percentage of gene-expressing cells in the first and the second indicated conditions (n=1). HG, High Glucose (11 mM); LG, Low Glucose (1mM).

Supplementary Figure 5
**(A, B)** UMAP representation of the pan-cancer scRNA-seq CD4^+^ T cell atlas colored by dataset name **(A)**, and sample type **(B)**. **(C)** Schematic representation of TXNIP isoforms as well as sgRNAs and primers used to validate them for CRISPR-Cas9 TXNIP KO.

Supplementary Figure 6
**(A)** Box plots of the indicated soluble factor concentrations in anti-PD-1-treated MLR, displaying group of numerical data through their 3^rd^ and 1^st^ quantiles (box), median (central band), minimum and maximum (whiskers) (n=6). Statistical analyses: Wilcoxon or T test, p-value is considered significantly relevant when p< 0.05 for the corresponding soluble factor (n=6). **(B)** Box plots of the mean fluorescent intensity (upper panel) or percentage (lower panel) for the indicated markers in anti-PD-1-treated MLR, displaying group of numerical data through their 3^rd^ and 1^st^ quantiles (box), median (central band), minimum and maximum (whiskers) (n=5). Statistical analyses: Wilcoxon or T test, p-value is considered significantly relevant when p<0.05 for the corresponding soluble factor. **(C)** Barplot of the percentage of CD4^+^ and CD8^+^ T cells in NT (non-transduced), NY WT (TCR only) and NY KO (TCR with TXNIP KO) T cells for the indicated donors. **(D)** Barplot of the percentage of TCR-expressing cells in NT (non-transduced), NY WT (TCR only) and NY KO (TCR with TXNIP KO) T cells for the indicated donors. **(E, F)** Red Area Per Well of NY-ESO-1^+^ A375 tumor cells was plotted over time alone or after addition of NT (non-transduced), NY WT (TCR only) and NY KO (TCR with TXNIP KO) T cells for donor 3 (D3) **(E)** or donor 4 (D4) **(F)**.

Supplementary Figure 7 Schematic representation of proposed hypothetical mechanism of action of TXNIP in immunometabolism and T cell activation. In low glucose condition or when TXNIP is decreased/depleted, either Glut-1 expression is enhanced and aerobic glycolysis is increased, and/or PTEN is inactivated by TRX leading to enhanced PI3K/Akt signaling and increased IFN-γ secretion.

Supplementary Table 1 Differential expression analysis results supporting **Figures 3A, B**.
