# Supplementary figures and images for: Glucose deprivation and identification of TXNIP as an immunometabolic modulator of T cell activation in cancer
Source: Front Immunol. 2025 Apr 7;16:1548509. doi: 10.3389/fimmu.2025.1548509 (PMC12010123; doi:10.3389/fimmu.2025.1548509)

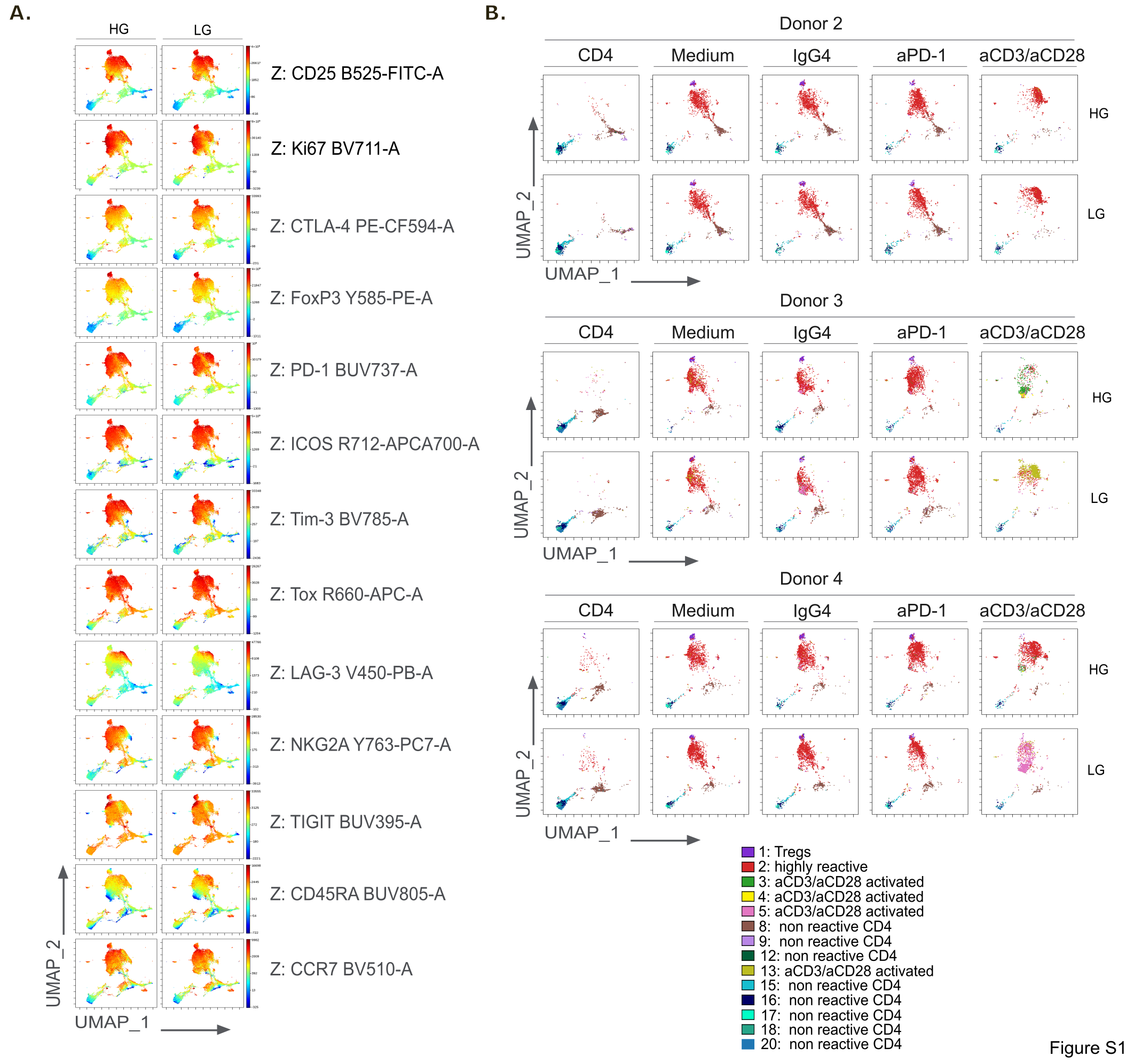

Supplement: Supplementary Figure 1 — (A) Concatenated flow cytometry UMAP dimensionality reduction representation of CD4+ T cells under high glucose (HG) or low glucose (LG) colored by indicated markers’ mean fluorescent intensity for all donors (n=4). (B) Individual flow cytometry UMAP dimensionality reduction representation of CD4+ T cells upon MLR stimulation colored by non-supervised clustering (Donor 2, Donor 3 and Donor 4). HG, High Glucose (11 mM); LG, Low Glucose (1mM). [file Image1.tiff]

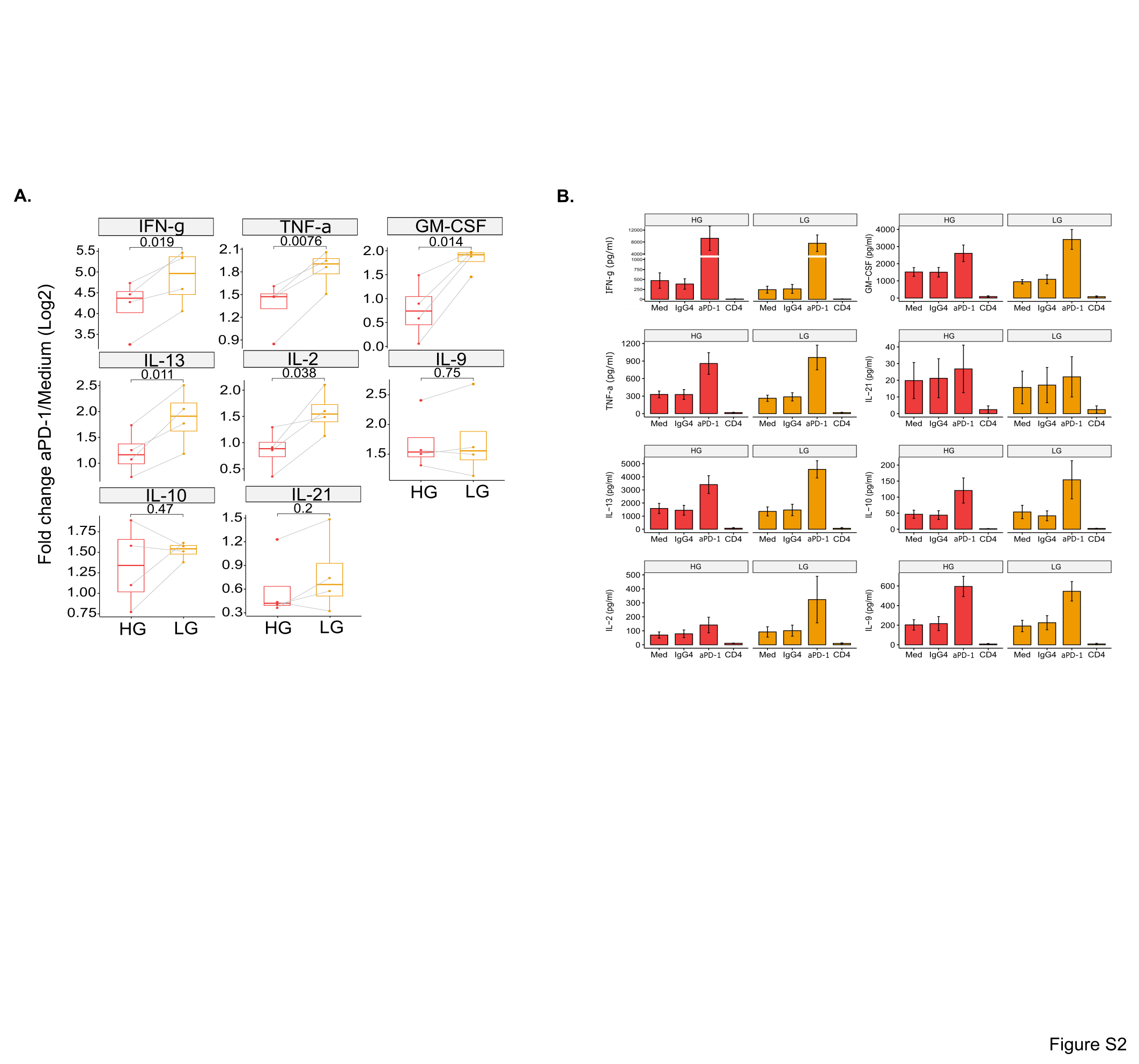

Supplement: Supplementary Figure 2 — (A) Box plots of the Log2 fold change (FC) of each cytokine and chemokine concentration in anti-PD-1-treated MLR over untreated MLR (Medium), displaying group of numerical data through their 3rd and 1st quantiles (box), median (central band), minimum and maximum (whiskers) (n=4). Statistical analyses: p-value is considered significantly relevant when p<0.05 for the corresponding soluble factor. (B) Barplot of indicated soluble factor concentrations in untreated (Med), isotype control (IgG4), anti-PD-1-treated (aPD-1) MLR or CD4+ T cells alone (CD4) under high glucose (HG, 11 mM) or low glucose (LG, 1 mM) conditions. [file Image2.tiff]

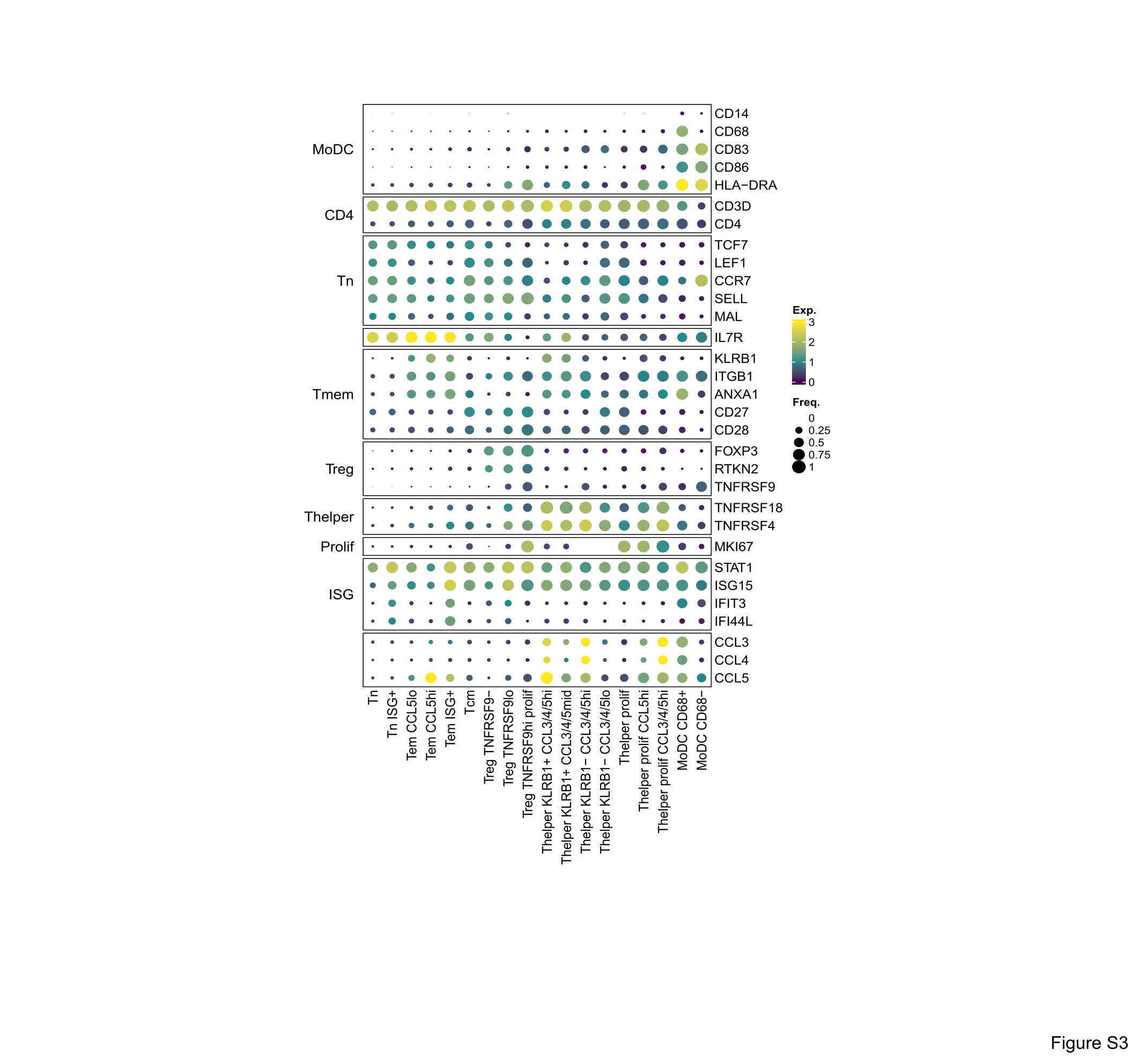

Supplement: Supplementary Figure 3 — Bubble plot showing expression of representative signature genes of the in vitro scRNA-seq data. Color represents the normalized expression level and size represents the expression frequency (n=1). [file Image3.tiff]

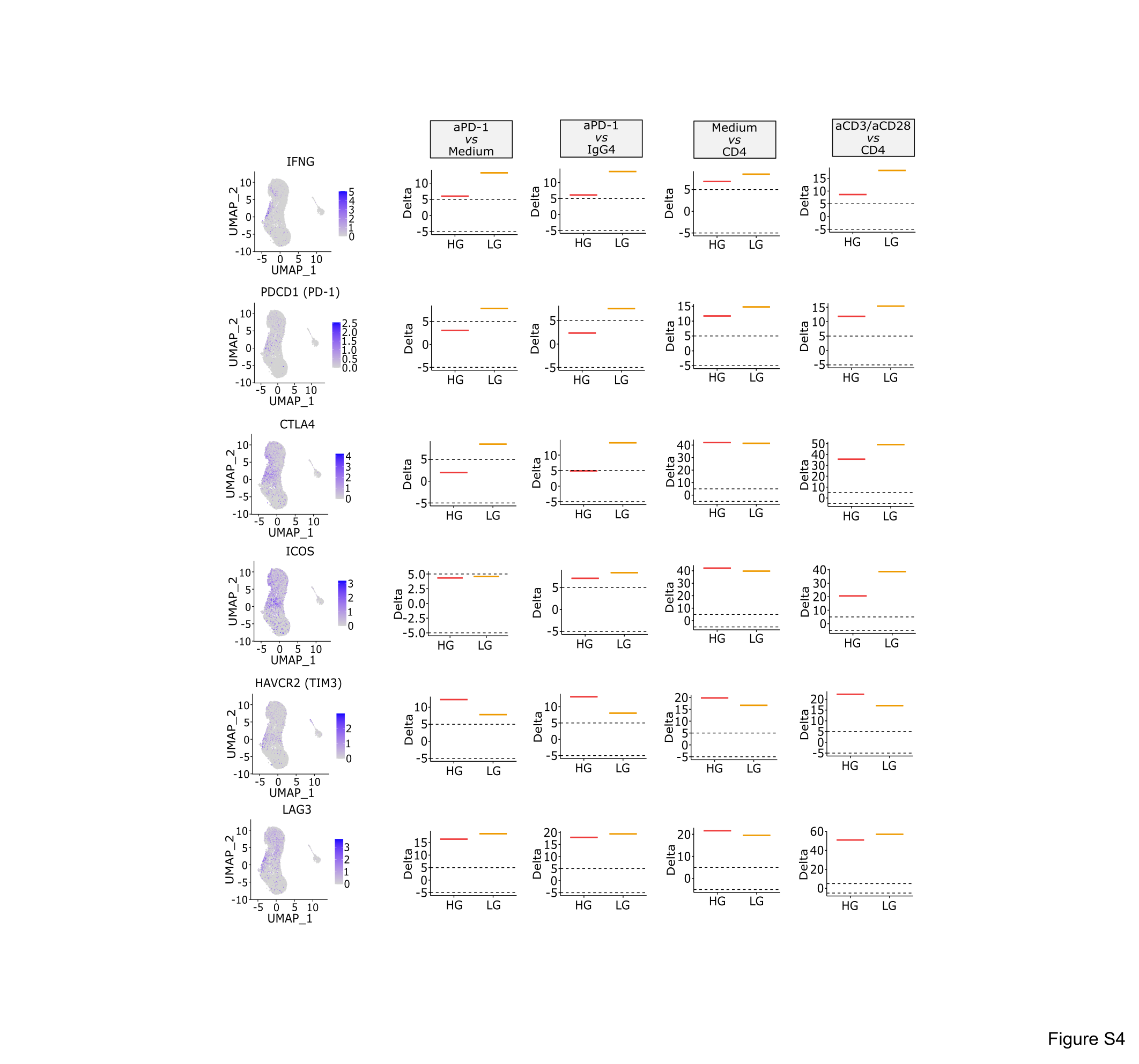

Supplement: Supplementary Figure 4 — UMAP representation of RNA expression and box plot of differential percentage of expressing cells for the indicated genes. Delta represents the difference between the percentage of gene-expressing cells in the first and the second indicated conditions (n=1). HG, High Glucose (11 mM); LG, Low Glucose (1mM). [file Image4.tiff]

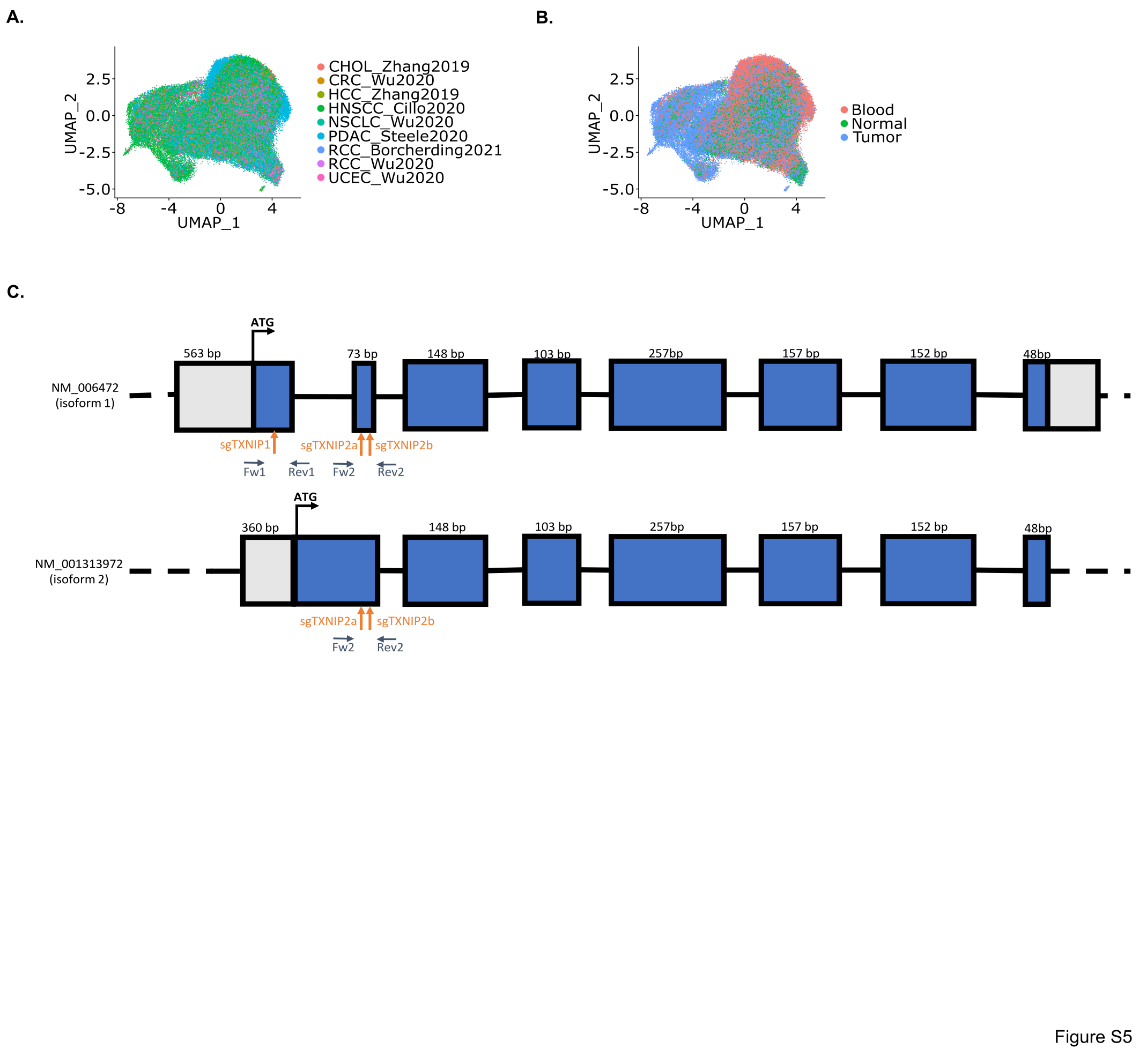

Supplement: Supplementary Figure 5 — (A, B) UMAP representation of the pan-cancer scRNA-seq CD4+ T cell atlas colored by dataset name (A), and sample type (B). (C) Schematic representation of TXNIP isoforms as well as sgRNAs and primers used to validate them for CRISPR-Cas9 TXNIP KO. [file Image5.tiff]

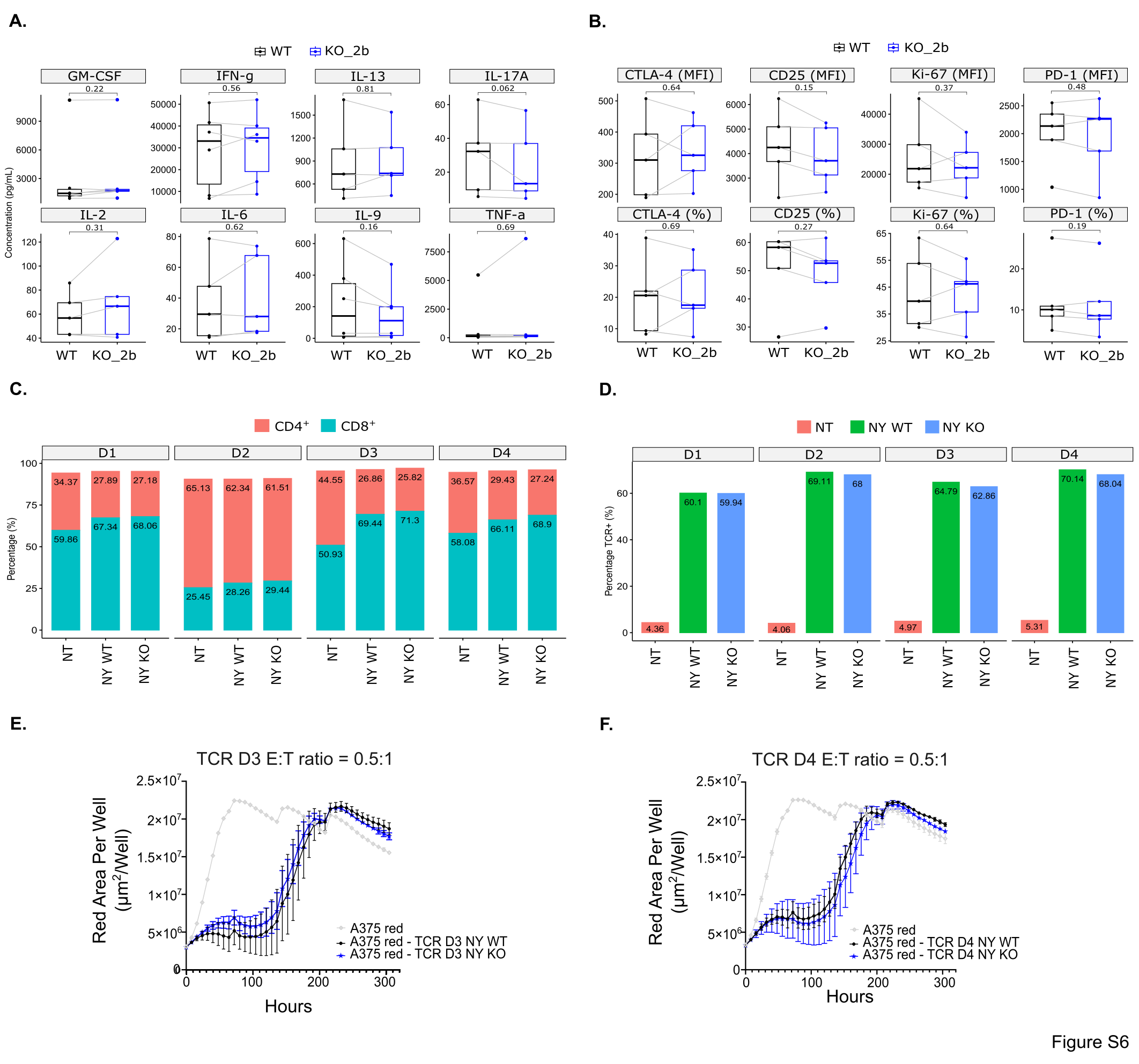

Supplement: Supplementary Figure 6 — (A) Box plots of the indicated soluble factor concentrations in anti-PD-1-treated MLR, displaying group of numerical data through their 3rd and 1st quantiles (box), median (central band), minimum and maximum (whiskers) (n=6). Statistical analyses: Wilcoxon or T test, p-value is considered significantly relevant when p< 0.05 for the corresponding soluble factor (n=6). (B) Box plots of the mean fluorescent intensity (upper panel) or percentage (lower panel) for the indicated markers in anti-PD-1-treated MLR, displaying group of numerical data through their 3rd and 1st quantiles (box), median (central band), minimum and maximum (whiskers) (n=5). Statistical analyses: Wilcoxon or T test, p-value is considered significantly relevant when p<0.05 for the corresponding soluble factor. (C) Barplot of the percentage of CD4+ and CD8+ T cells in NT (non-transduced), NY WT (TCR only) and NY KO (TCR with TXNIP KO) T cells for the indicated donors. (D) Barplot of the percentage of TCR-expressing cells in NT (non-transduced), NY WT (TCR only) and NY KO (TCR with TXNIP KO) T cells for the indicated donors. (E, F) Red Area Per Well of NY-ESO-1+ A375 tumor cells was plotted over time alone or after addition of NT (non-transduced), NY WT (TCR only) and NY KO (TCR with TXNIP KO) T cells for donor 3 (D3) (E) or donor 4 (D4) (F). [file Image6.tiff]

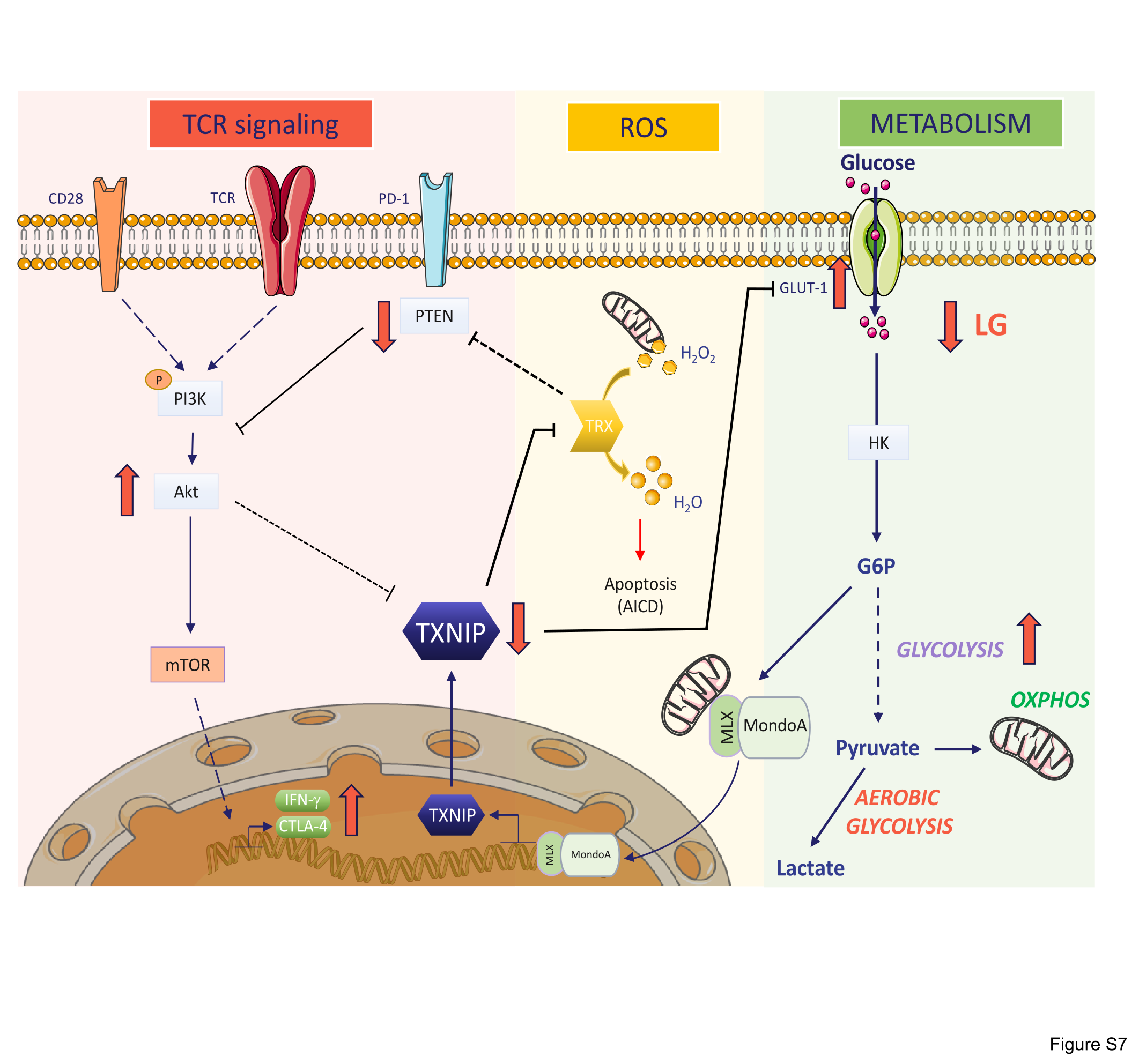

Supplement: Supplementary Figure 7 — Schematic representation of proposed hypothetical mechanism of action of TXNIP in immunometabolism and T cell activation. In low glucose condition or when TXNIP is decreased/depleted, either Glut-1 expression is enhanced and aerobic glycolysis is increased, and/or PTEN is inactivated by TRX leading to enhanced PI3K/Akt signaling and increased IFN-γ secretion. [file Image7.tiff]
